# Supplementary material for: Spatio-temporal dynamics of dengue in Brazil: Seasonal travelling waves and determinants of regional synchrony
Source: PLoS Negl Trop Dis. 2019 Apr 22;13(4):e0007012. doi: 10.1371/journal.pntd.0007012 (PMC6497439; doi:10.1371/journal.pntd.0007012)
Supplement: S1 Fig — (PDF) [file pntd.0007012.s001.pdf]

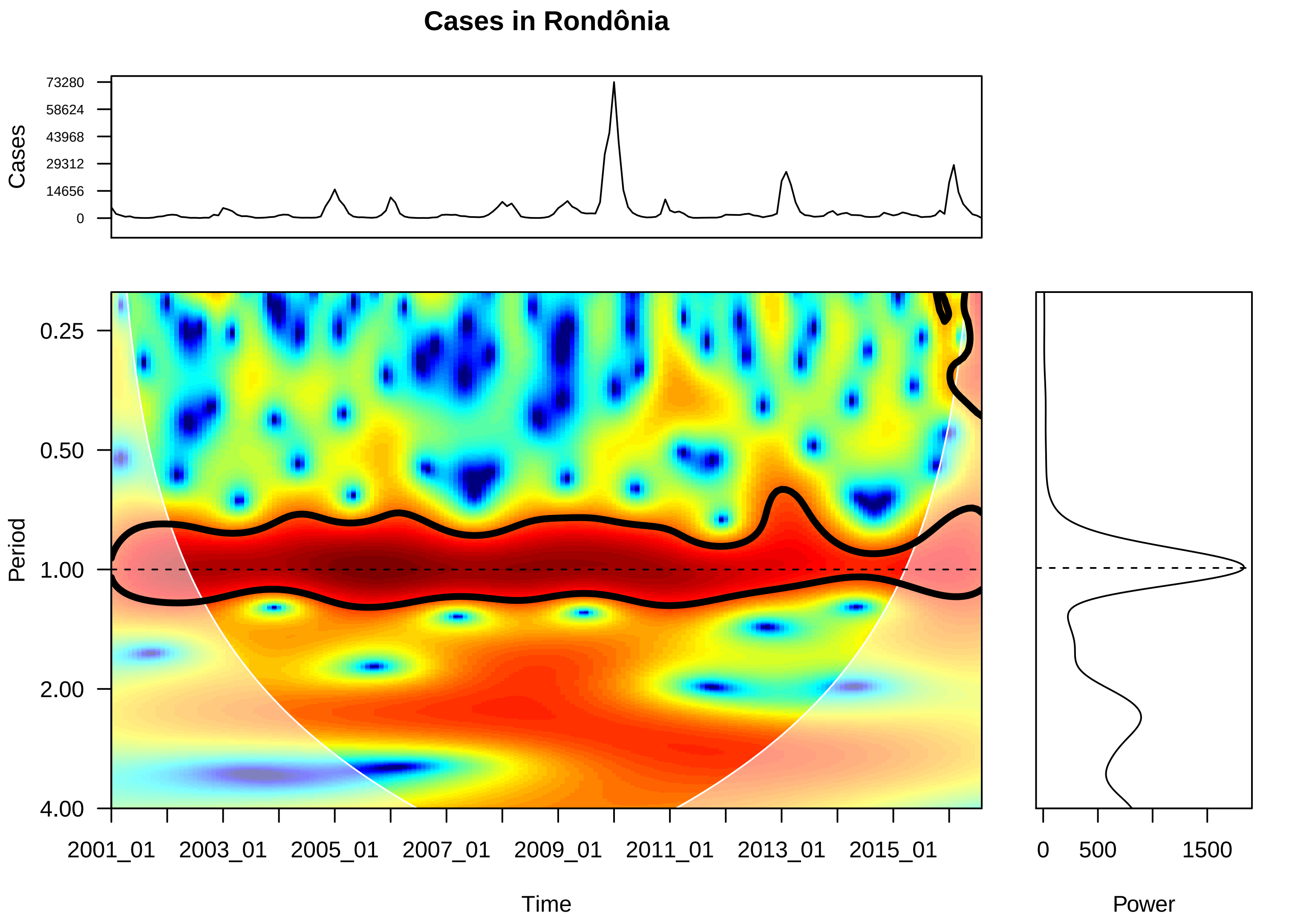

Cases in Rondônia
Cases
73280
58624
43968
29312
14656
0
Period
0.25
0.50
1.00
2.00
4.00
2001_01
2003_01
2005_01
2007_01
2009_01
2011_01
2013_01
2015_01
Time
0
500
1500
Power

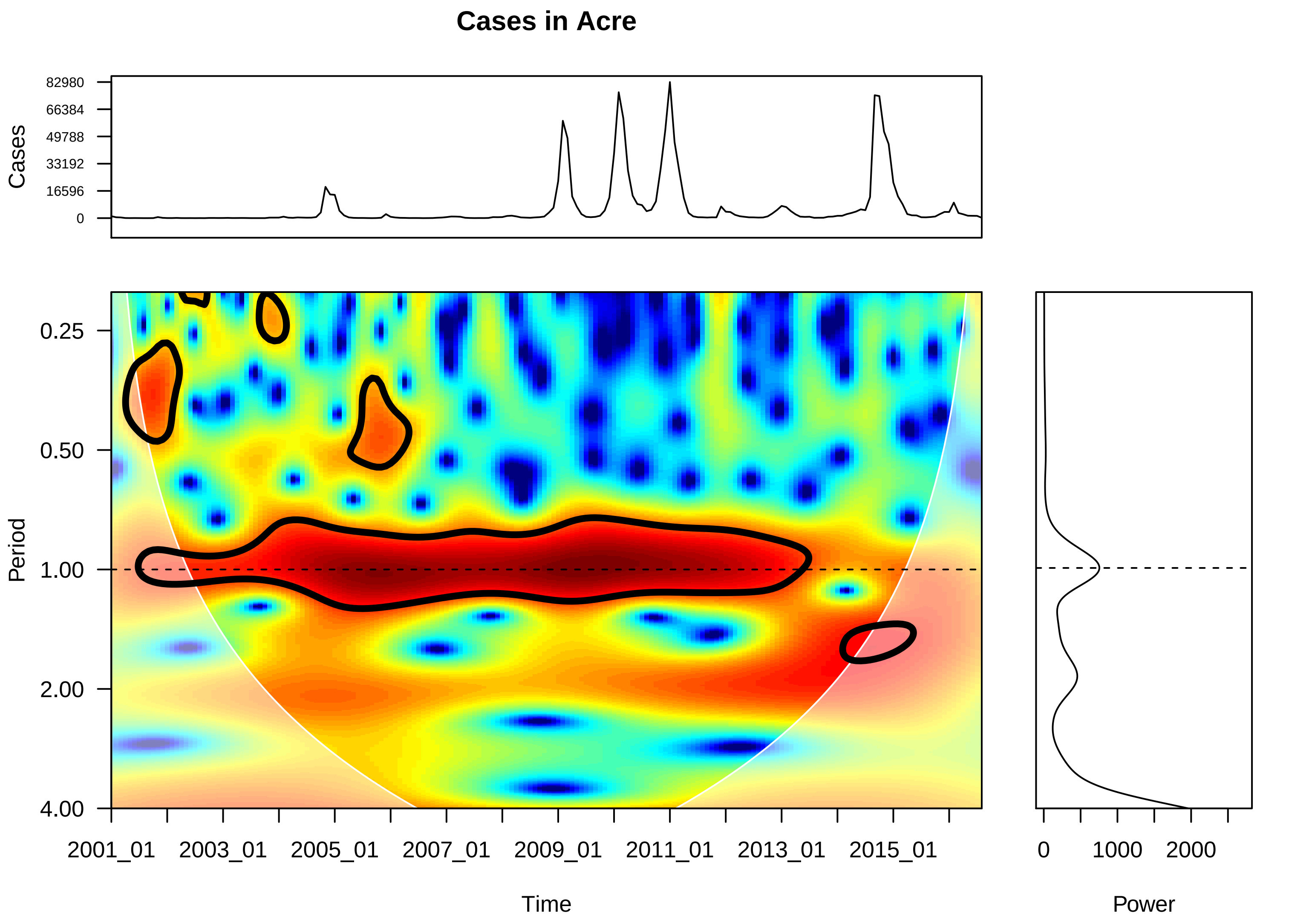

Cases in Acre
Cases
82980
66384
49788
33192
16596
0
Period
0.25
0.50
1.00
2.00
4.00
2001_01
2003_01
2005_01
2007_01
2009_01
2011_01
2013_01
2015_01
Time
0
1000
2000
Power

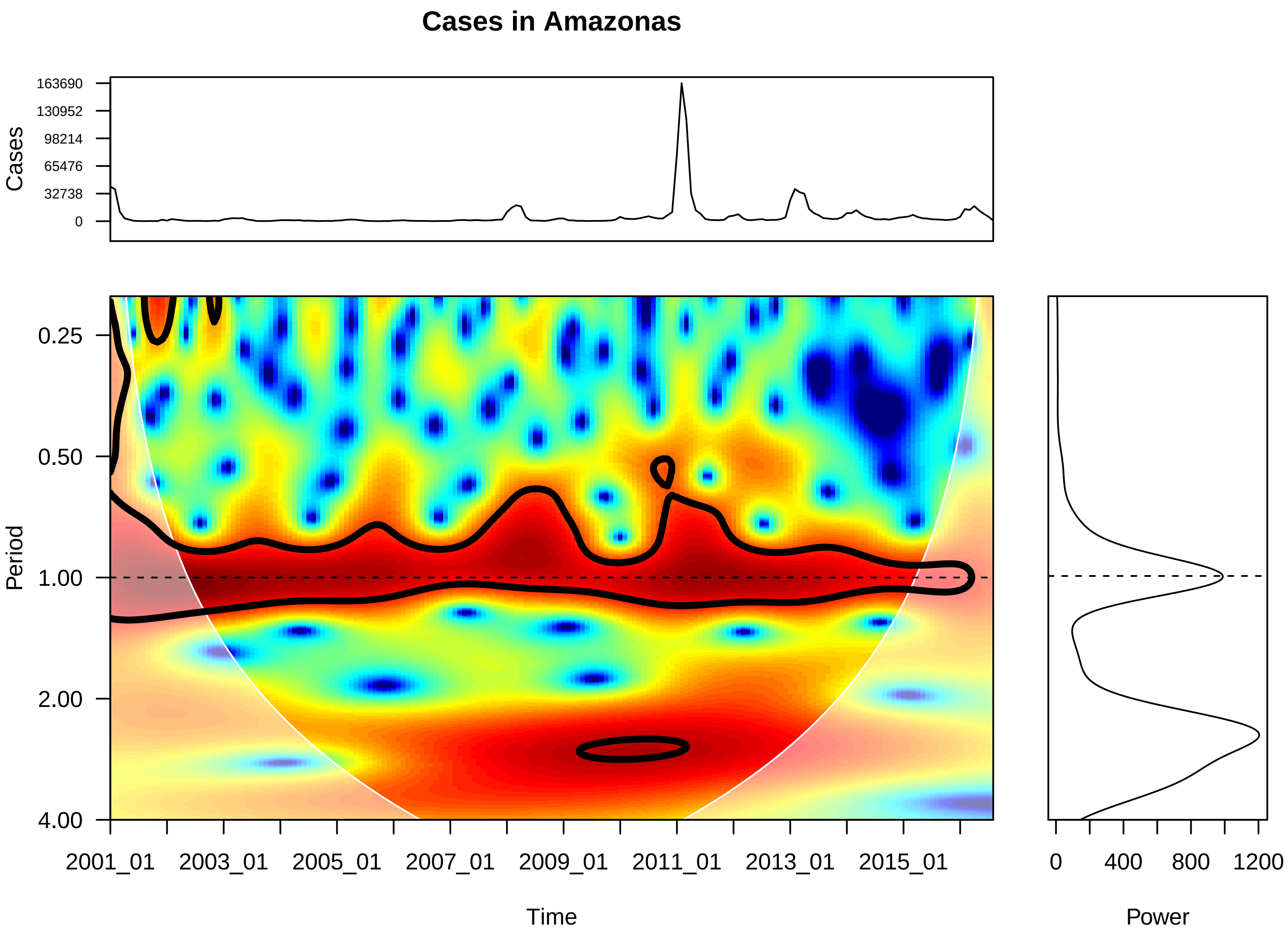
Cases in Amazonas
Cases
163690
130952
98214
65476
32738
0
Period
0.25
0.50
1.00
2.00
4.00
2001_01
2003_01
2005_01
2007_01
2009_01
2011_01
2013_01
2015_01
Time
0
400
800
1200
Power

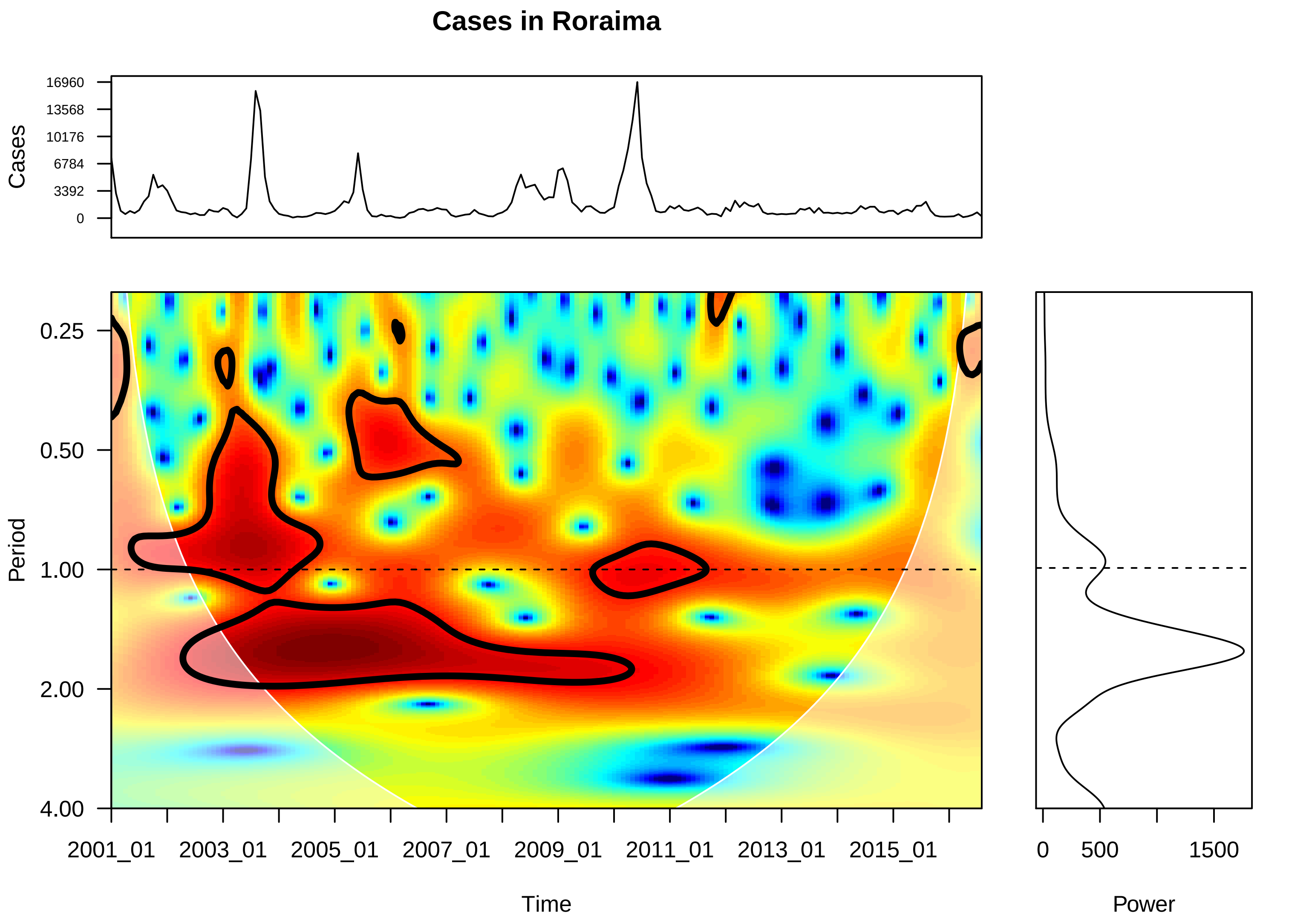

Cases in Roraima
Cases
16960
13568
10176
6784
3392
0
Period
0.25
0.50
1.00
2.00
4.00
2001_01
2003_01
2005_01
2007_01
2009_01
2011_01
2013_01
2015_01
Time
0
500
1500
Power

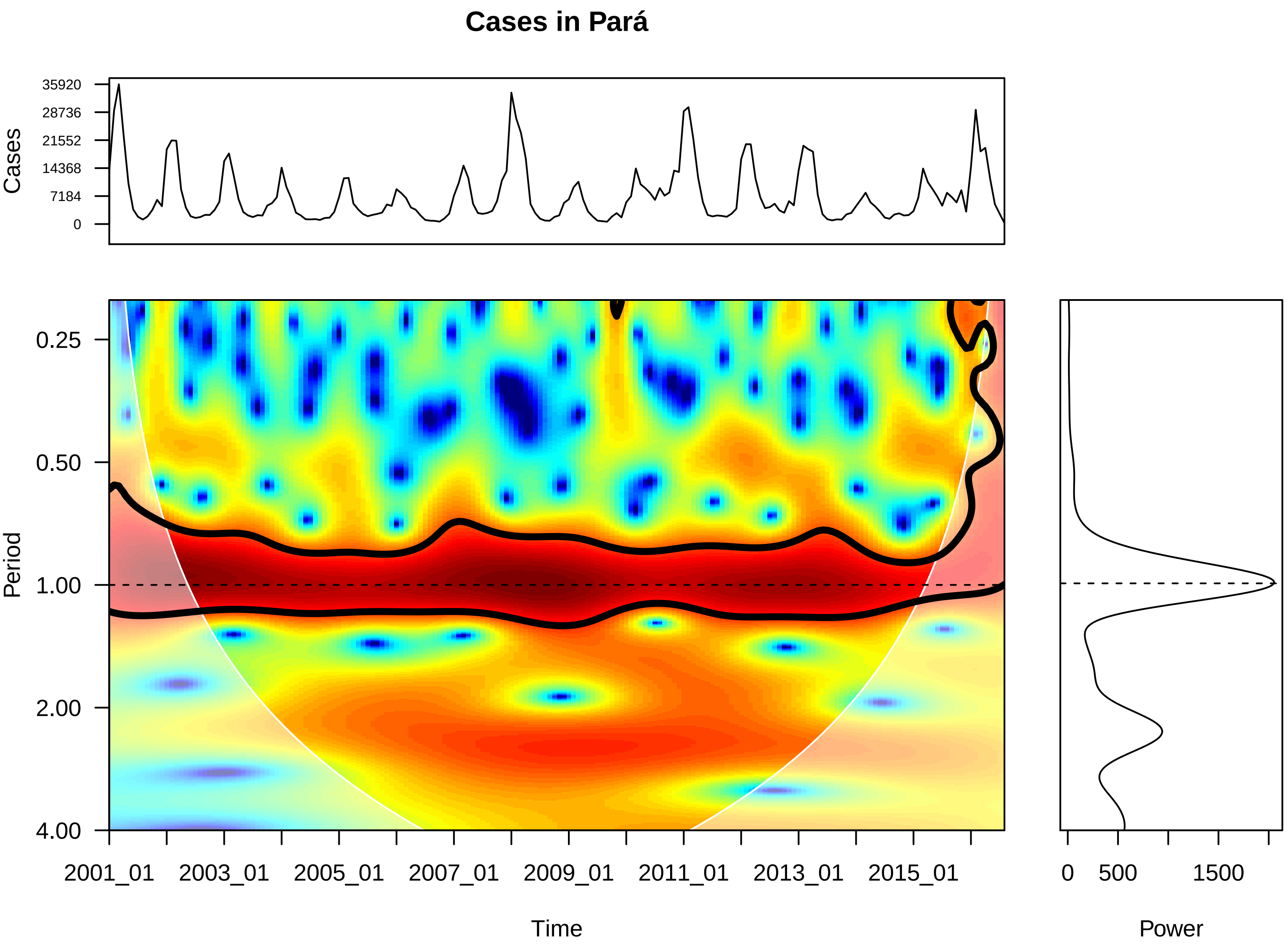
Cases in Pará
Cases
35920
28736
21552
14368
7184
0
Period
0.25
0.50
1.00
2.00
4.00
2001_01
2003_01
2005_01
2007_01
2009_01
2011_01
2013_01
2015_01
Time
0
500
1500
Power

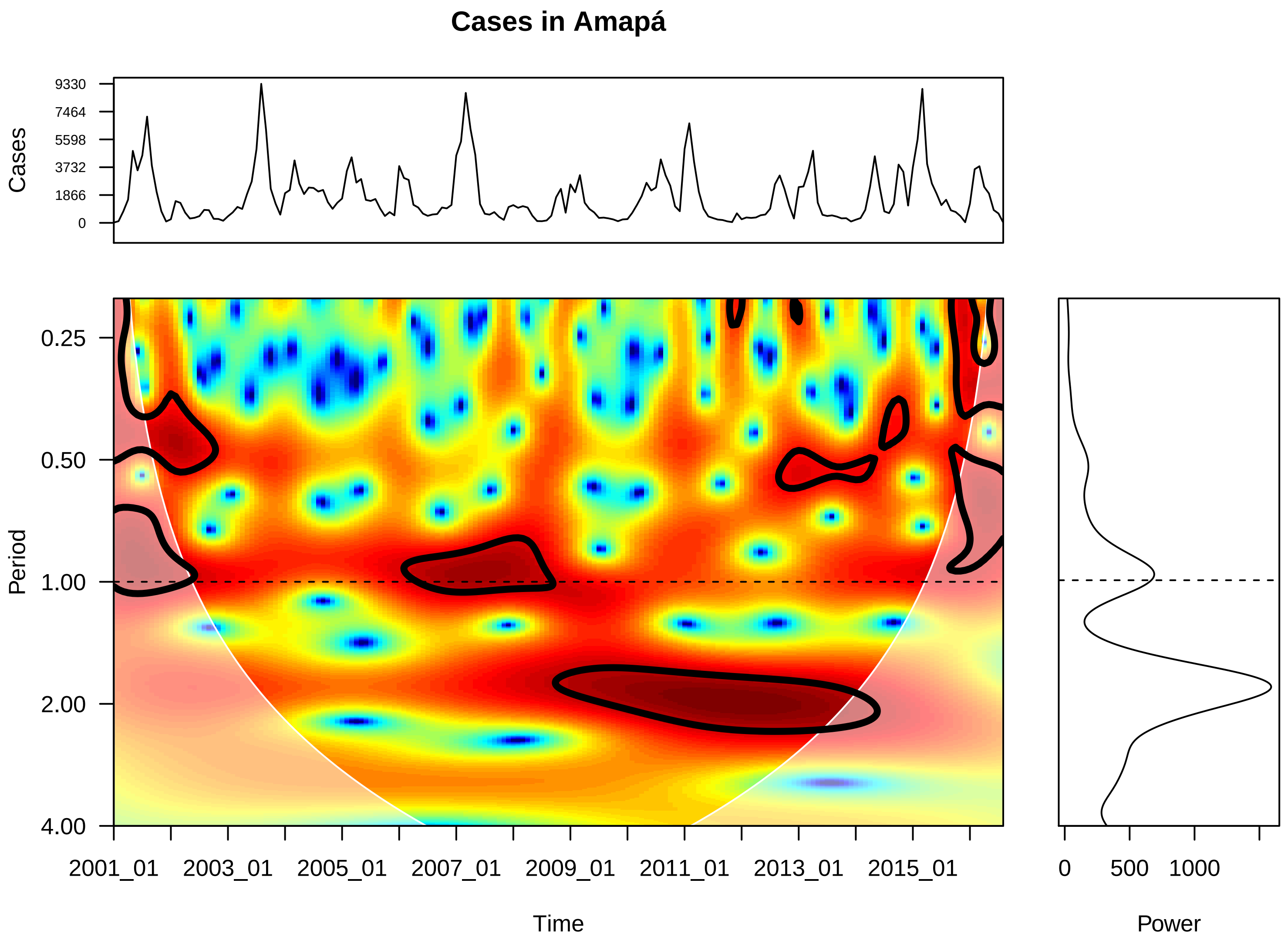
Cases in Amapá
Cases
9330
7464
5598
3732
1866
0
Period
0.25
0.50
1.00
2.00
4.00
2001_01
2003_01
2005_01
2007_01
2009_01
2011_01
2013_01
2015_01
Time
0
500
1000
Power

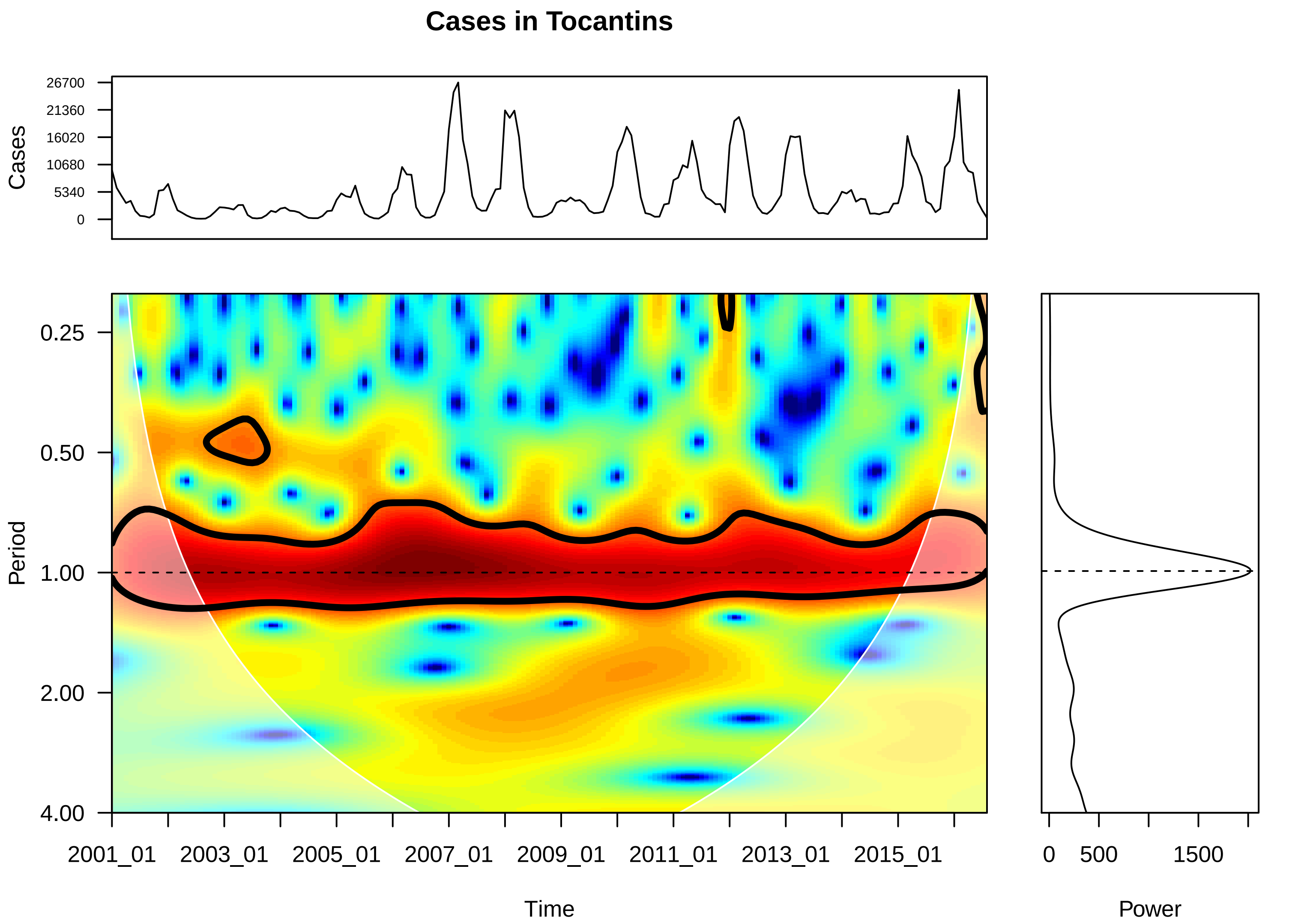

Cases in Tocantins
Cases
26700
21360
16020
10680
5340
0
Period
0.25
0.50
1.00
2.00
4.00
2001_01
2003_01
2005_01
2007_01
2009_01
2011_01
2013_01
2015_01
Time
0
500
1500
Power

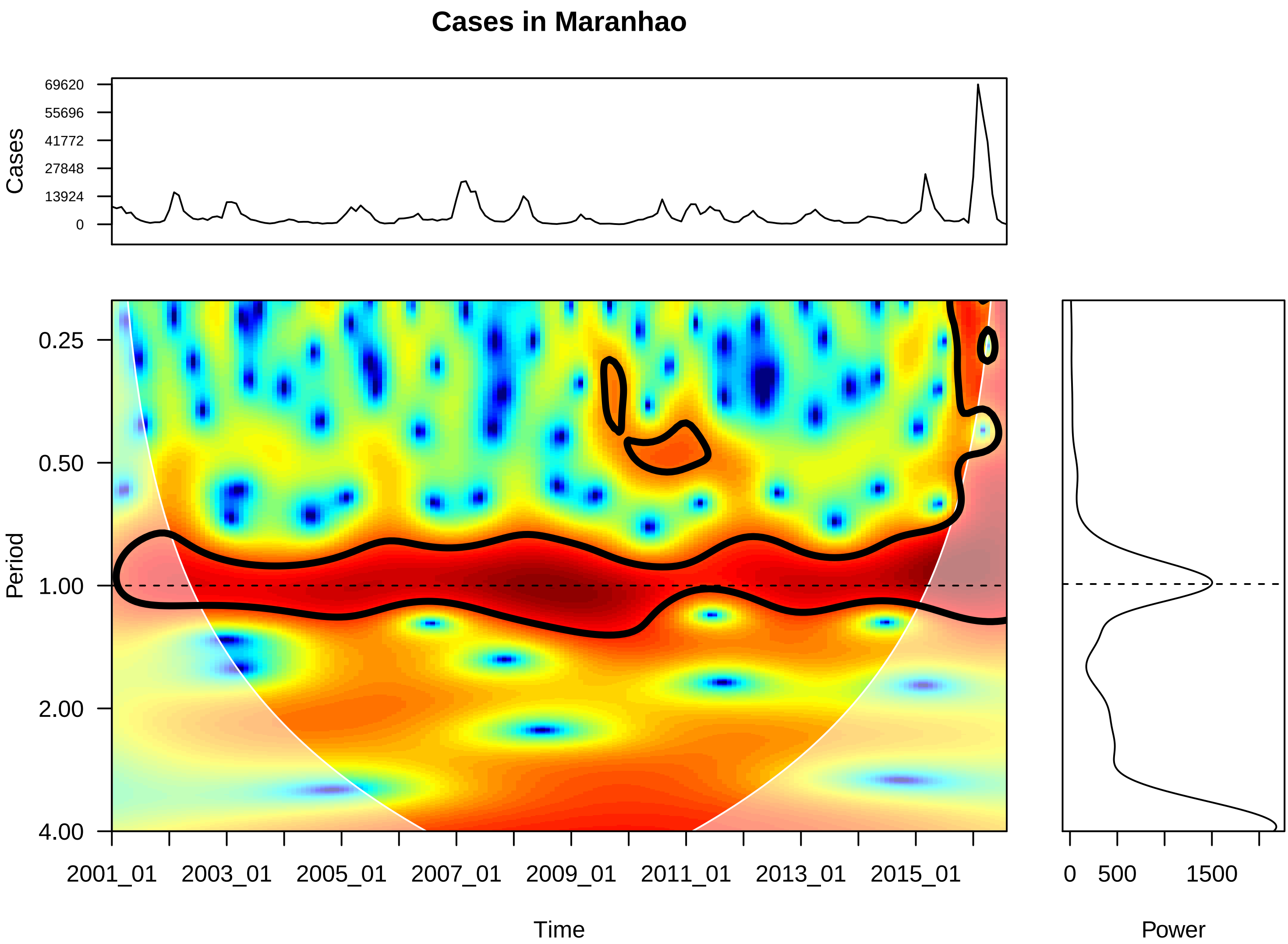

Cases in Maranhao
Cases
69620
55696
41772
27848
13924
0
Period
0.25
0.50
1.00
2.00
4.00
2001_01
2003_01
2005_01
2007_01
2009_01
2011_01
2013_01
2015_01
Time
0
500
1500
Power

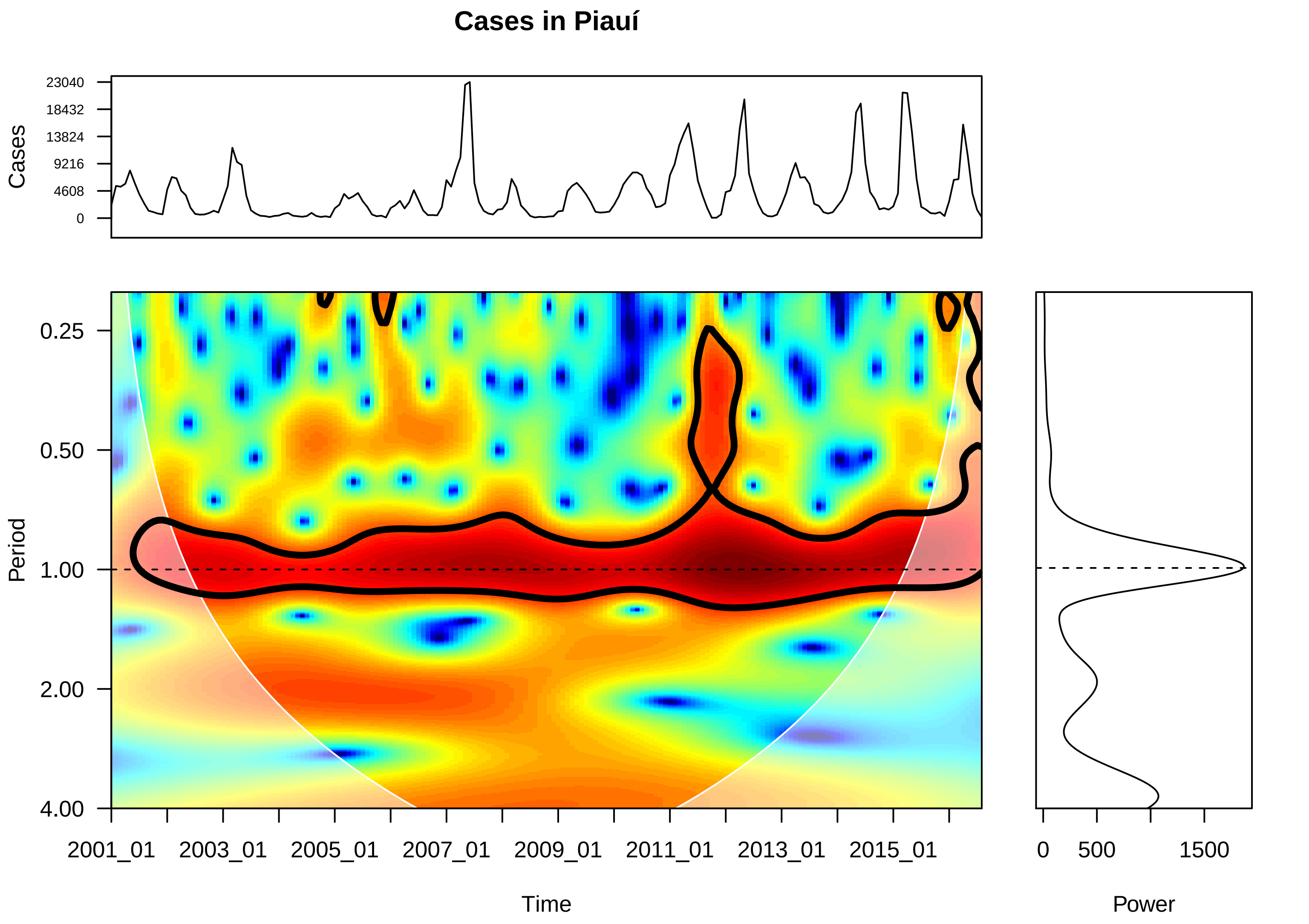
Cases in Piauí
Cases
23040
18432
13824
9216
4608
0
Period
0.25
0.50
1.00
2.00
4.00
2001_01
2003_01
2005_01
2007_01
2009_01
2011_01
2013_01
2015_01
Time
0
500
1500
Power

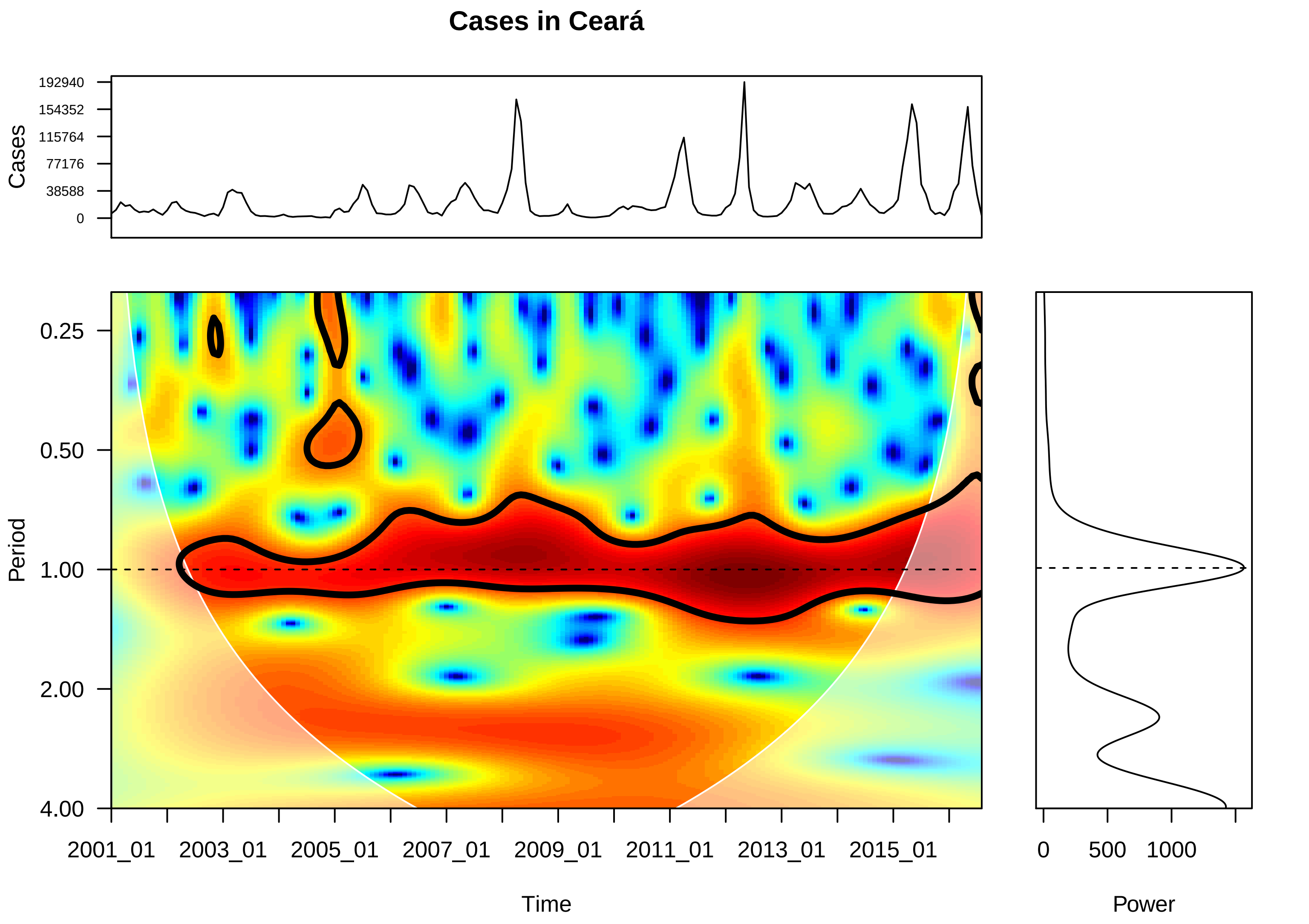

Cases in Ceará
Cases
192940
154352
115764
77176
38588
0
Period
0.25
0.50
1.00
2.00
4.00
2001_01
2003_01
2005_01
2007_01
2009_01
2011_01
2013_01
2015_01
Time
0
500
1000
Power

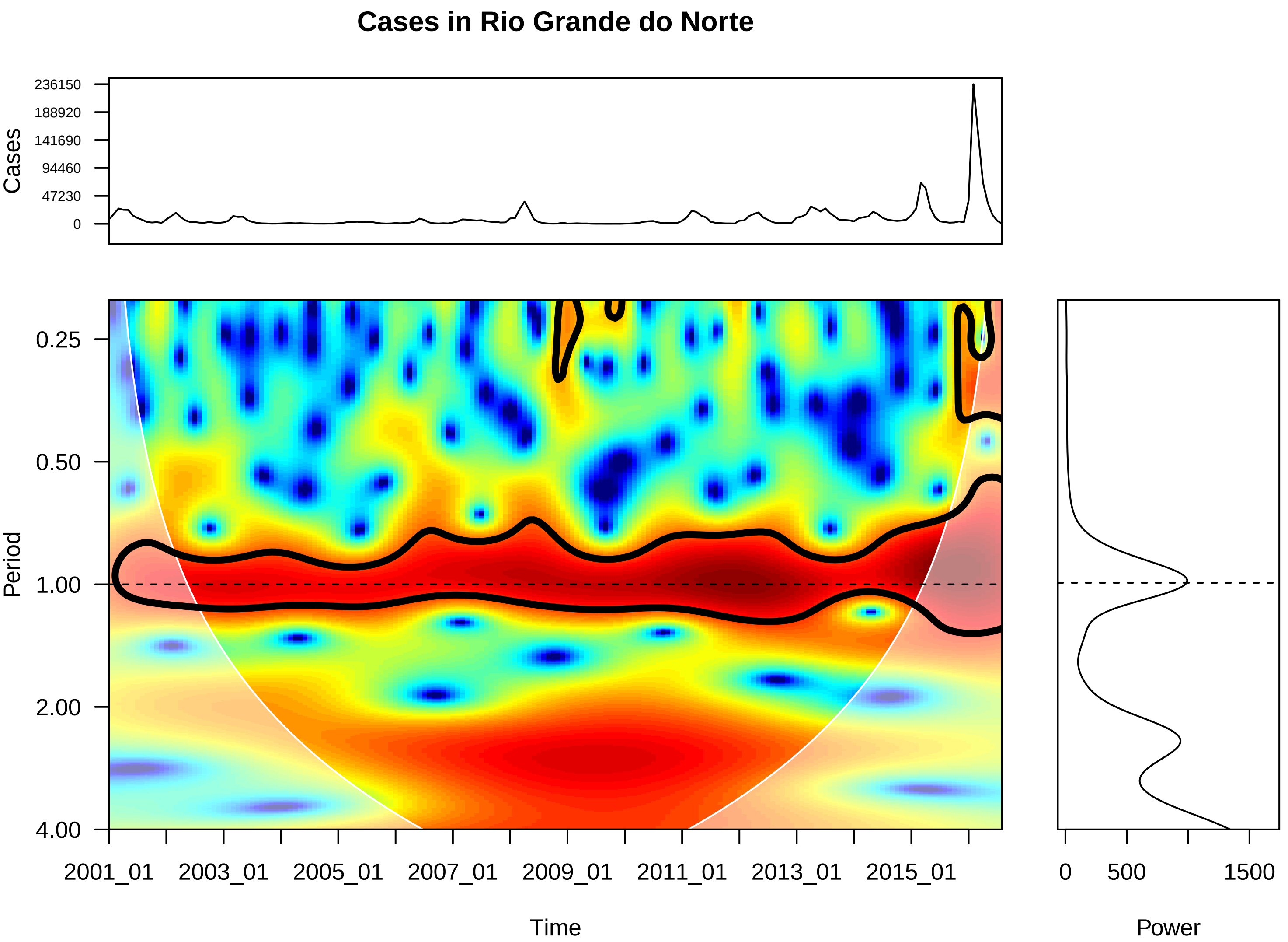

Cases in Rio Grande do Norte
Cases
236150
188920
141690
94460
47230
0
Period
0.25
0.50
1.00
2.00
4.00
2001_01
2003_01
2005_01
2007_01
2009_01
2011_01
2013_01
2015_01
Time
0
500
1500
Power

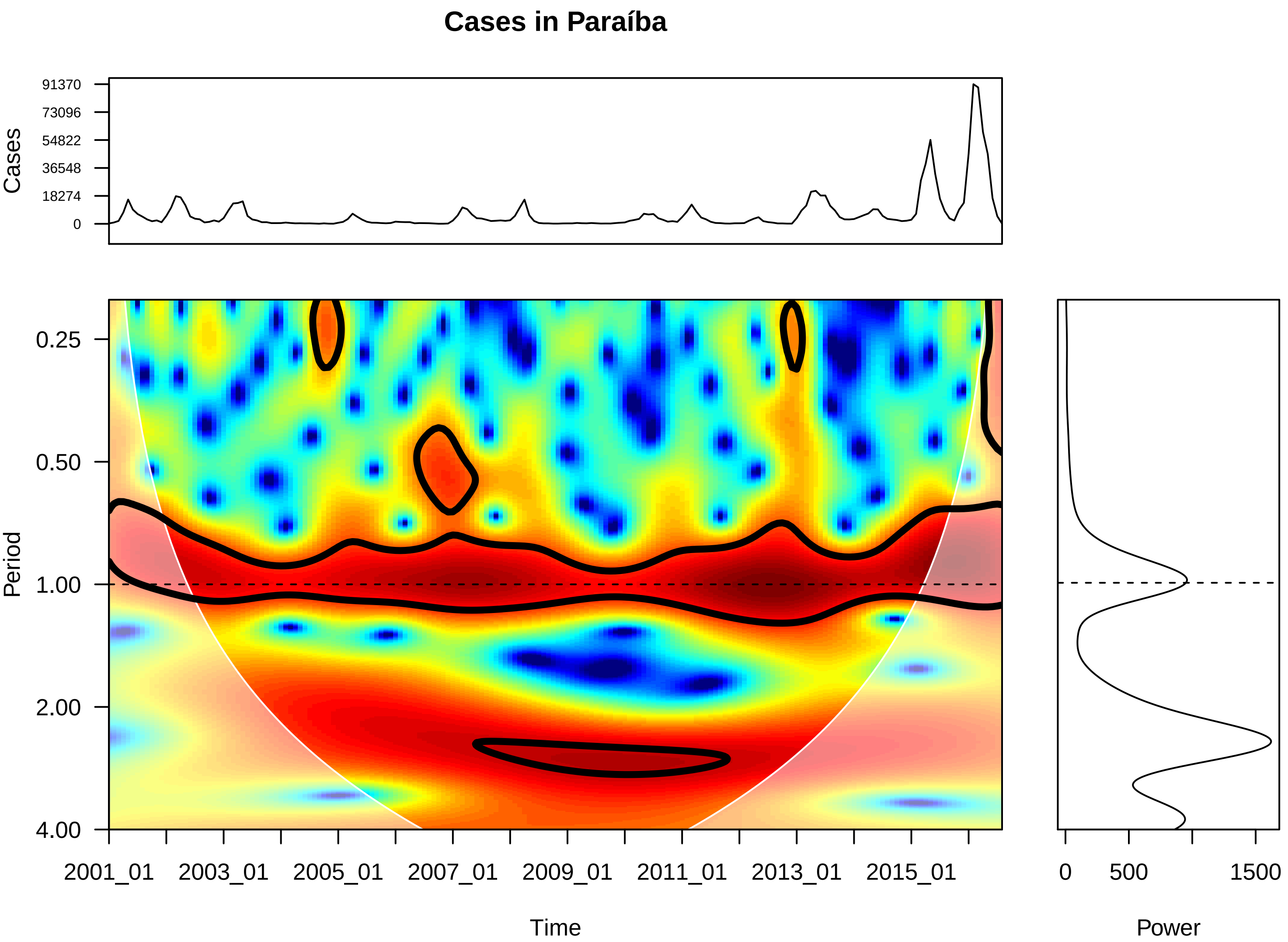

Cases in Paraíba
Cases
91370
73096
54822
36548
18274
0
Period
0.25
0.50
1.00
2.00
4.00
2001_01
2003_01
2005_01
2007_01
2009_01
2011_01
2013_01
2015_01
Time
0
500
1500
Power

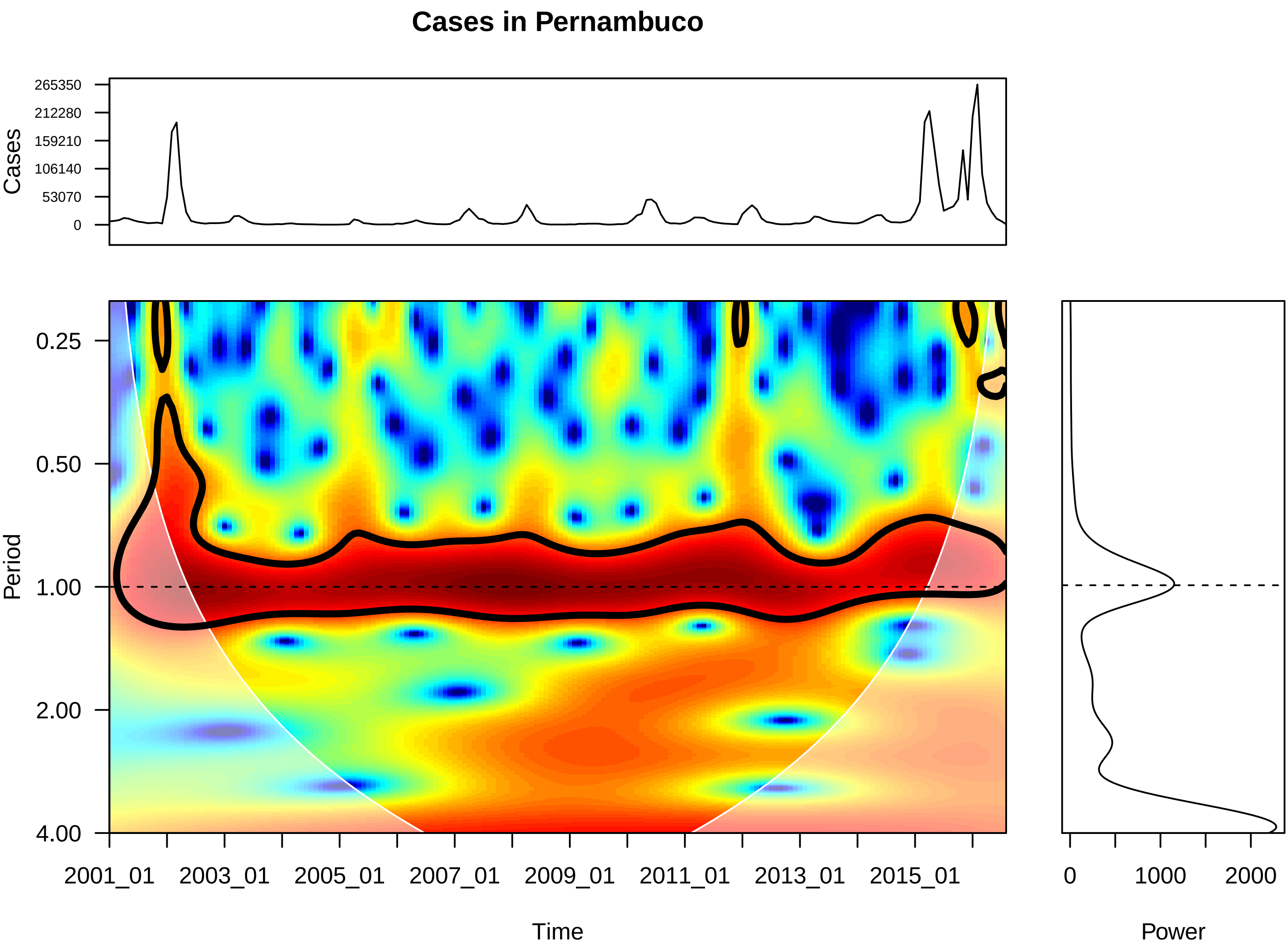

Cases in Pernambuco
Cases
265350
212280
159210
106140
53070
0
Period
0.25
0.50
1.00
2.00
4.00
2001_01
2003_01
2005_01
2007_01
2009_01
2011_01
2013_01
2015_01
Time
0
1000
2000
Power

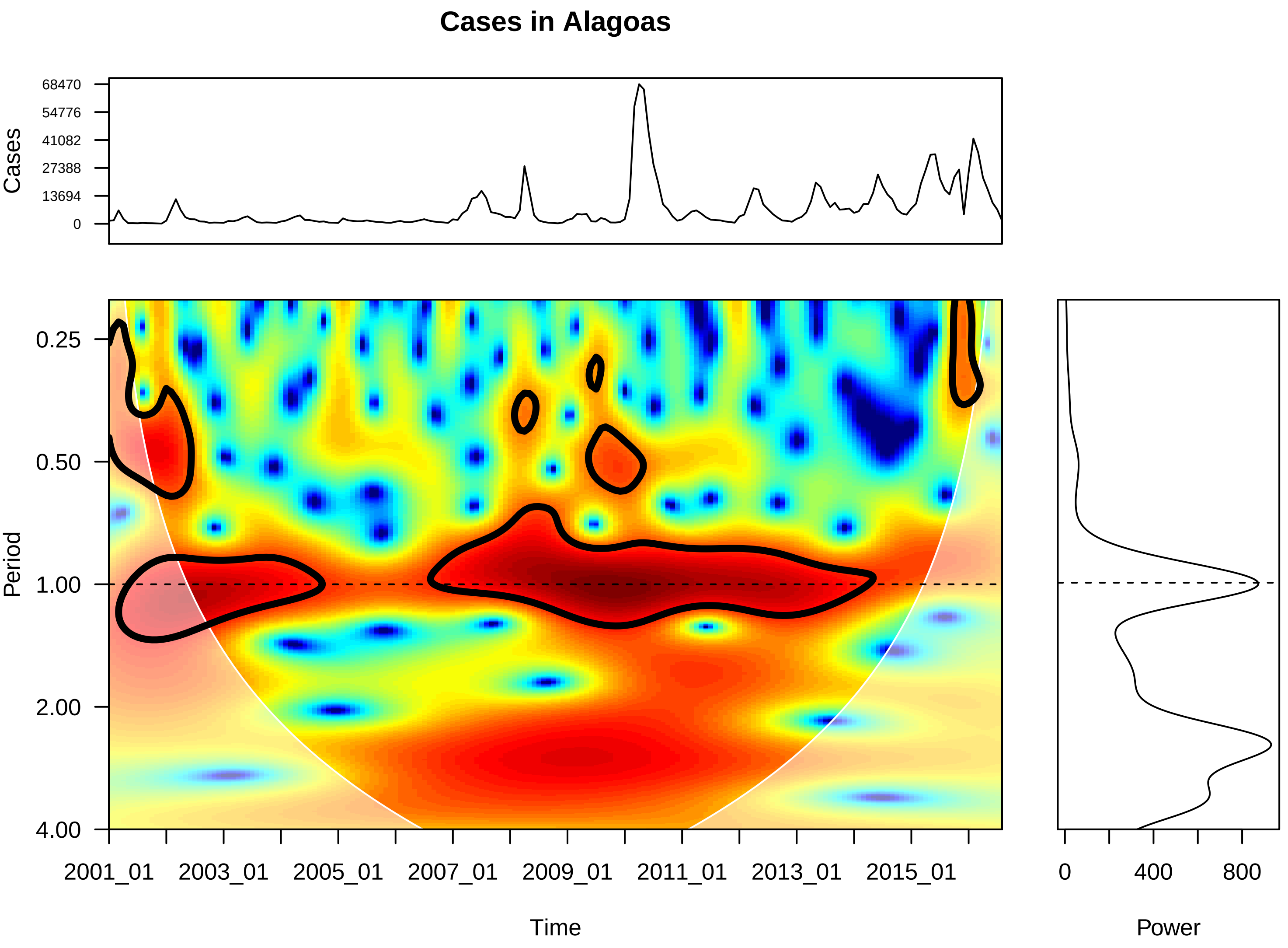
Cases in Alagoas
Cases
68470
54776
41082
27388
13694
0
Period
0.25
0.50
1.00
2.00
4.00
2001_01
2003_01
2005_01
2007_01
2009_01
2011_01
2013_01
2015_01
Time
0
400
800
Power

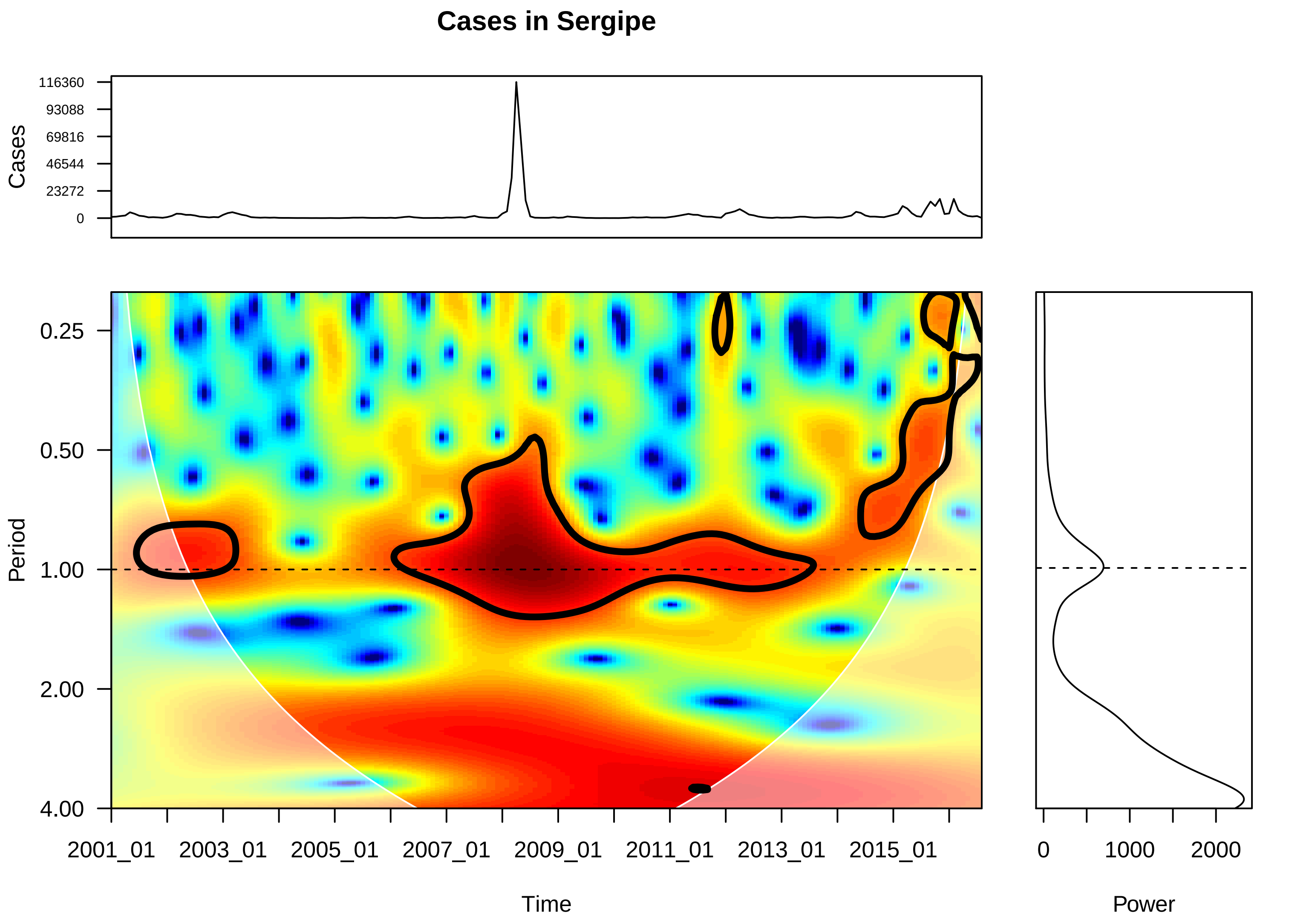

Cases in Sergipe
Cases
116360
93088
69816
46544
23272
0
Period
0.25
0.50
1.00
2.00
4.00
2001_01
2003_01
2005_01
2007_01
2009_01
2011_01
2013_01
2015_01
Time
0
1000
2000
Power

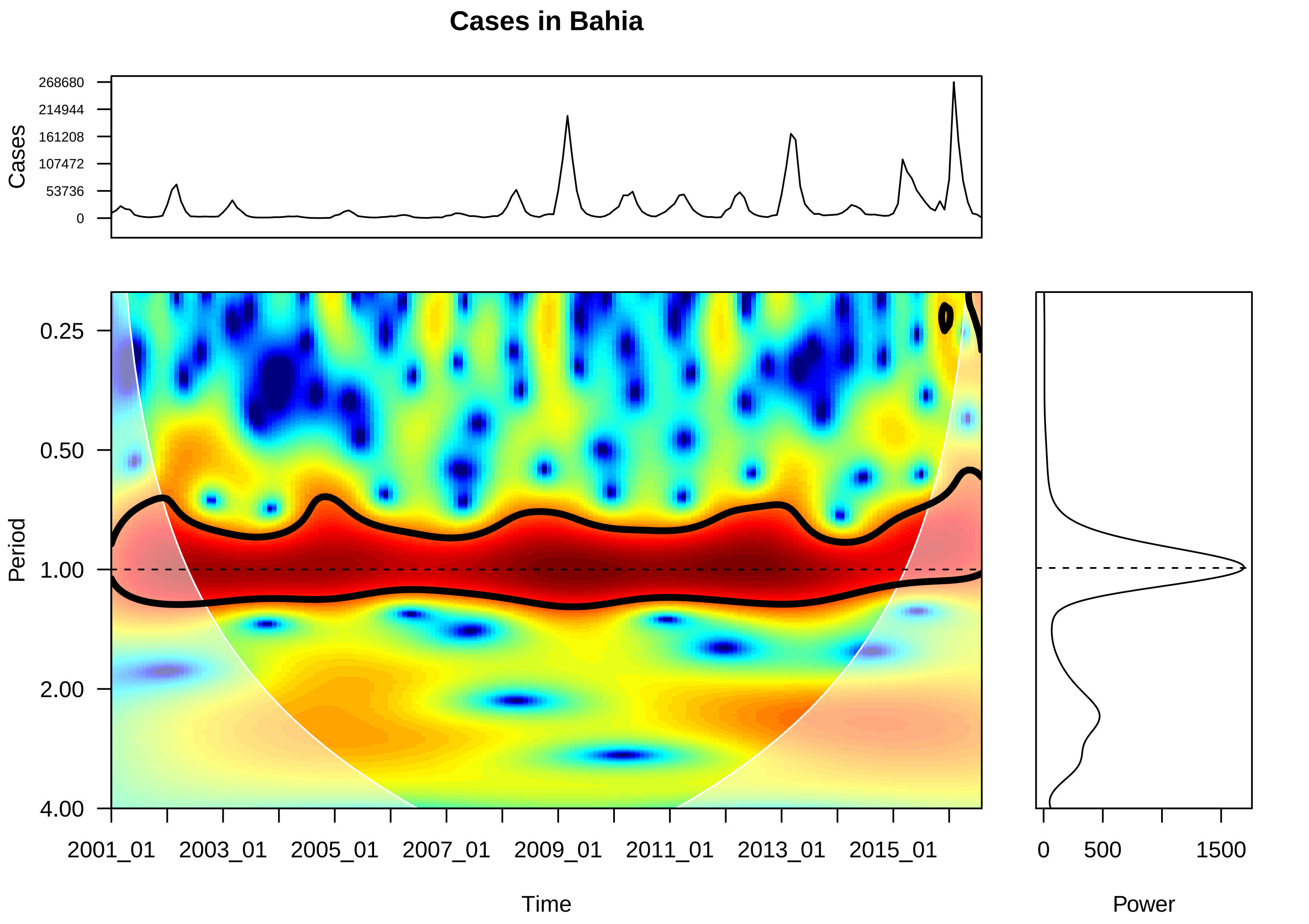

Cases in Bahia
Cases
268680
214944
161208
107472
53736
0
Period
0.25
0.50
1.00
2.00
4.00
2001_01
2003_01
2005_01
2007_01
2009_01
2011_01
2013_01
2015_01
Time
0
500
1500
Power

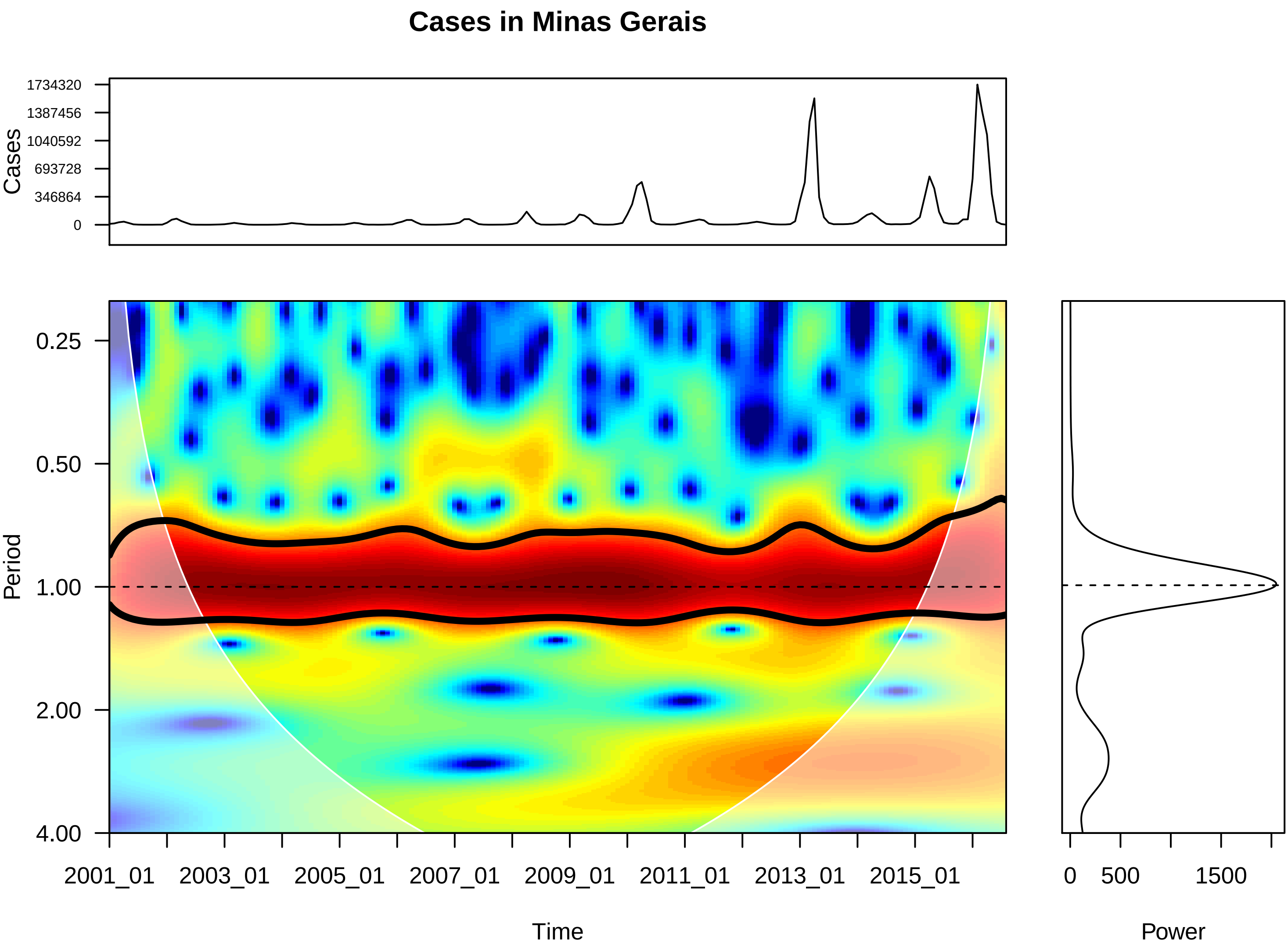
Cases in Minas Gerais
Cases
1734320
1387456
1040592
693728
346864
0
Period
0.25
0.50
1.00
2.00
4.00
2001_01
2003_01
2005_01
2007_01
2009_01
2011_01
2013_01
2015_01
Time
0
500
1500
Power

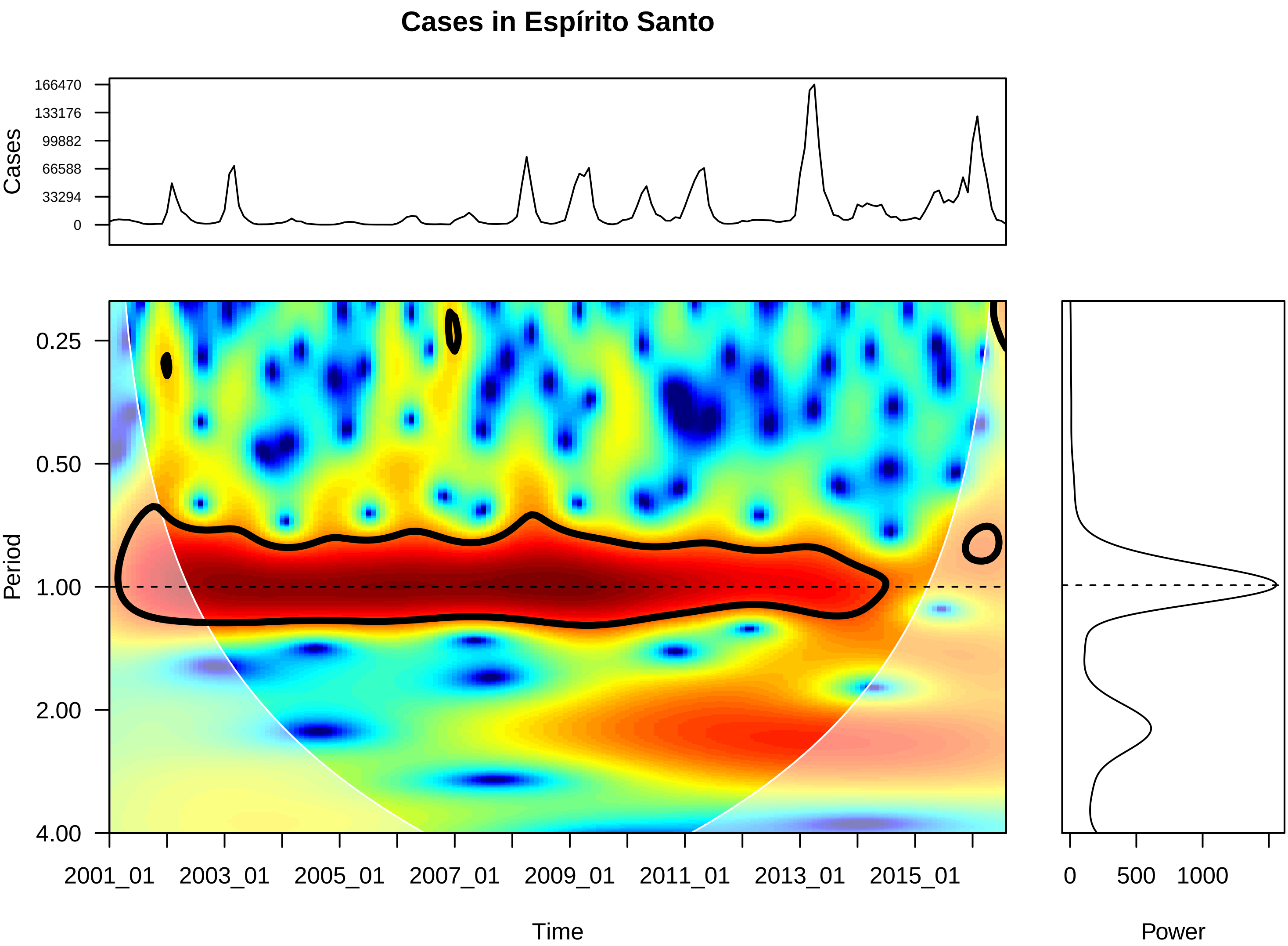

Cases in Espírito Santo
Cases
166470
133176
99882
66588
33294
0
Period
0.25
0.50
1.00
2.00
4.00
2001_01
2003_01
2005_01
2007_01
2009_01
2011_01
2013_01
2015_01
Time
0
500
1000
Power

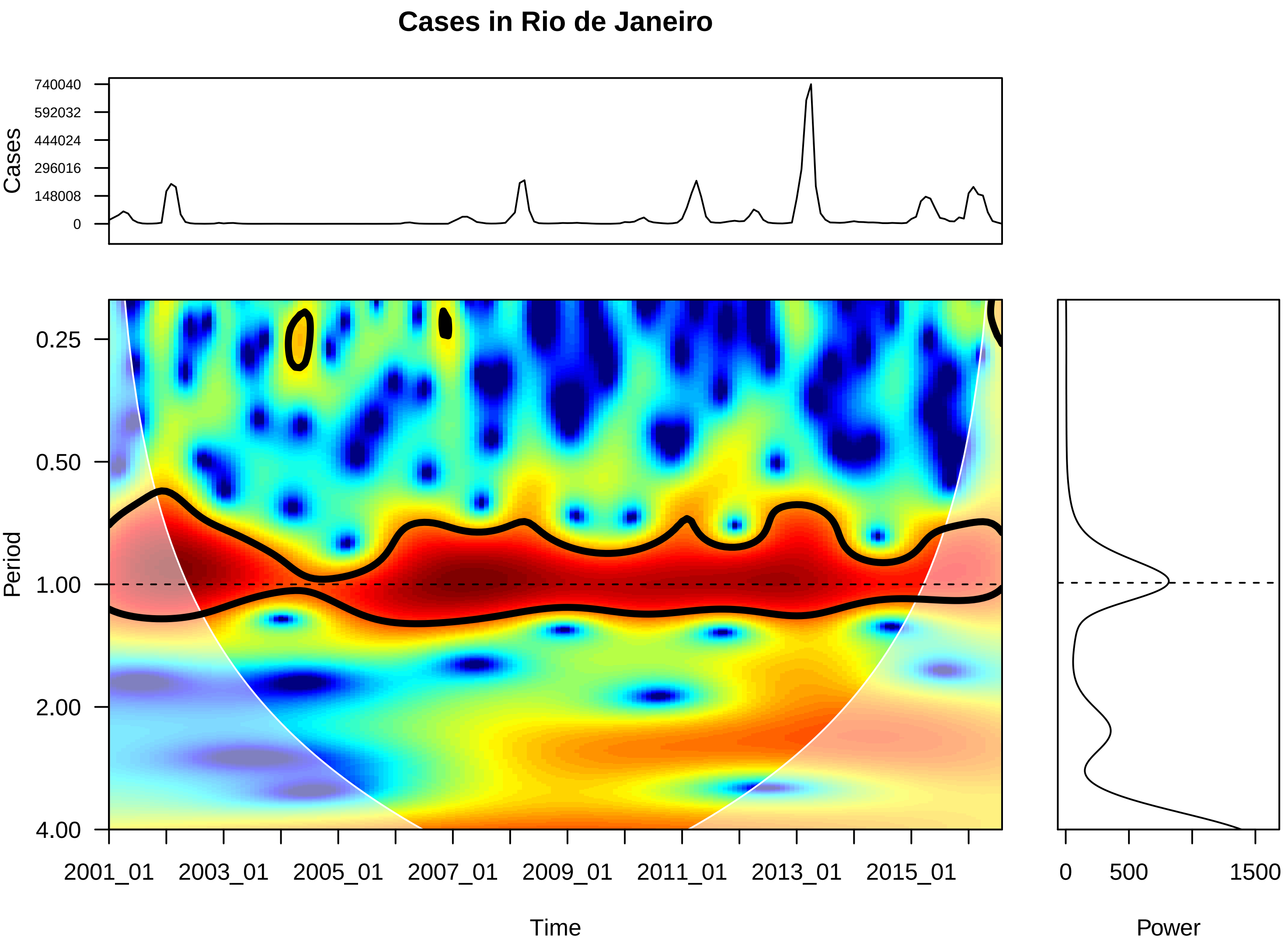
Cases in Rio de Janeiro
Cases
740040
592032
444024
296016
148008
0
Period
0.25
0.50
1.00
2.00
4.00
2001_01
2003_01
2005_01
2007_01
2009_01
2011_01
2013_01
2015_01
Time
0
500
1500
Power

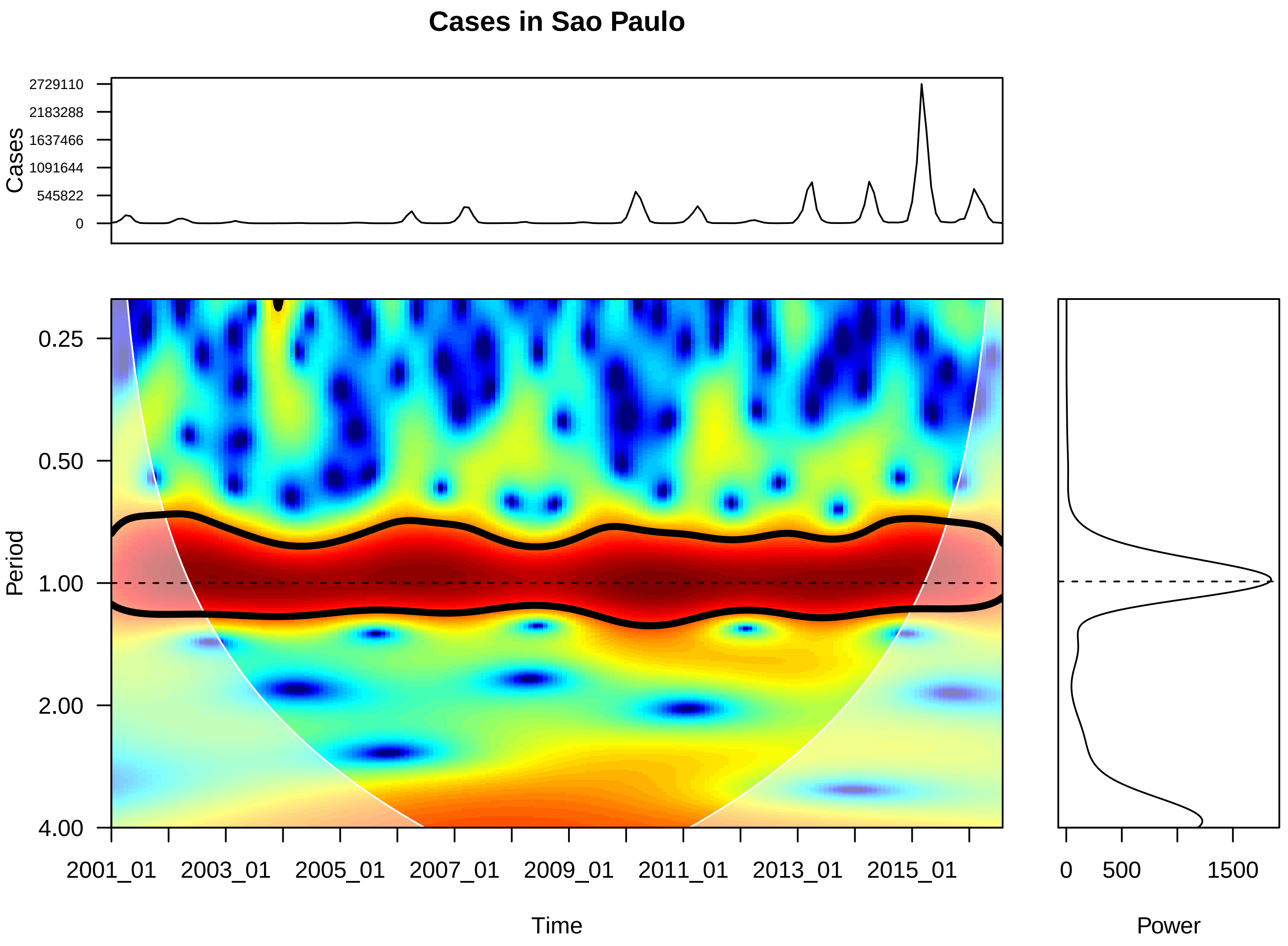

Cases in Sao Paulo
Cases
2729110
2183288
1637466
1091644
545822
0
Period
0.25
0.50
1.00
2.00
4.00
2001_01
2003_01
2005_01
2007_01
2009_01
2011_01
2013_01
2015_01
Time
0
500
1500
Power

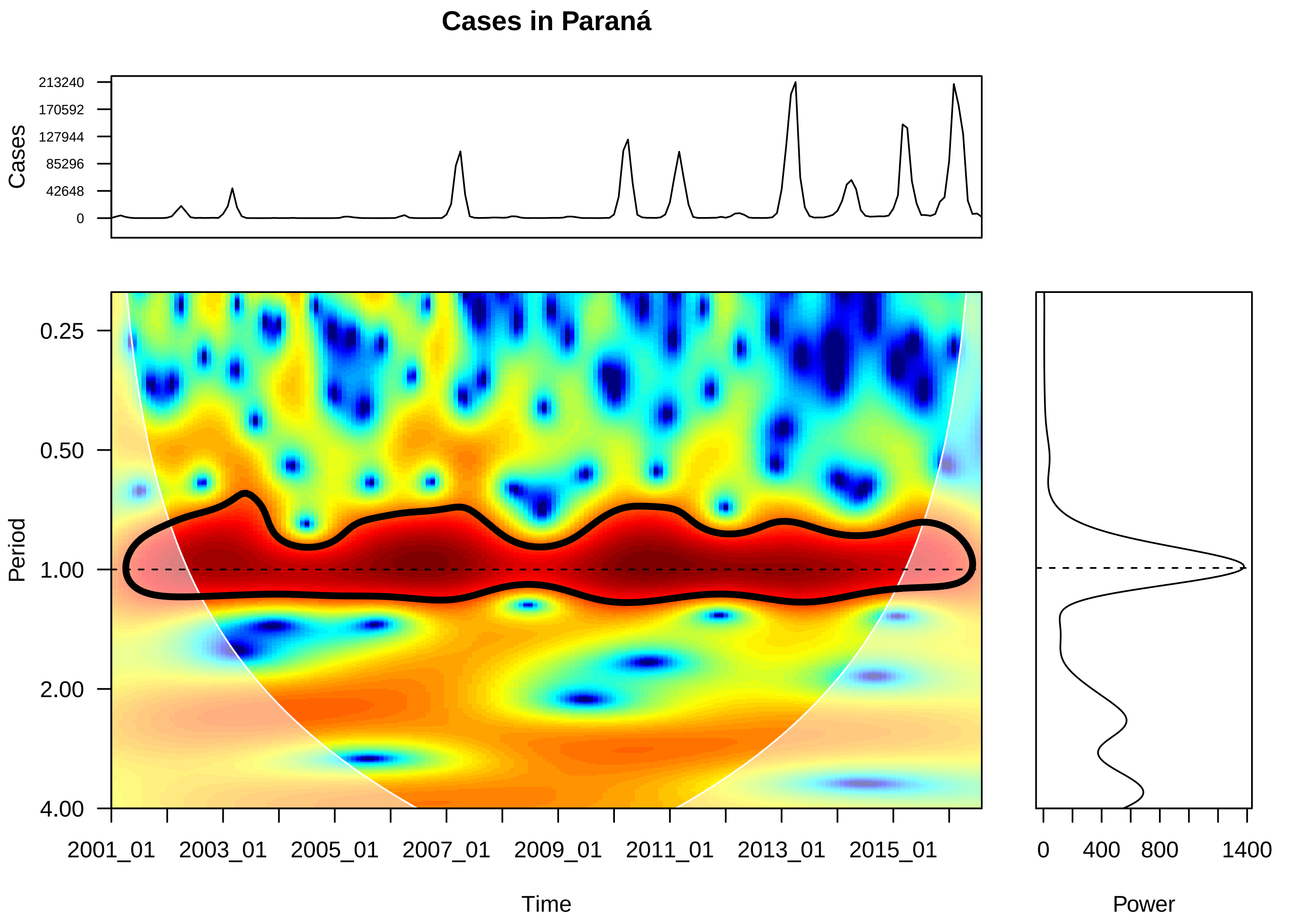
Cases in Paraná
Cases
213240
170592
127944
85296
42648
0
Period
0.25
0.50
1.00
2.00
4.00
2001_01
2003_01
2005_01
2007_01
2009_01
2011_01
2013_01
2015_01
Time
0
400
800
1400
Power

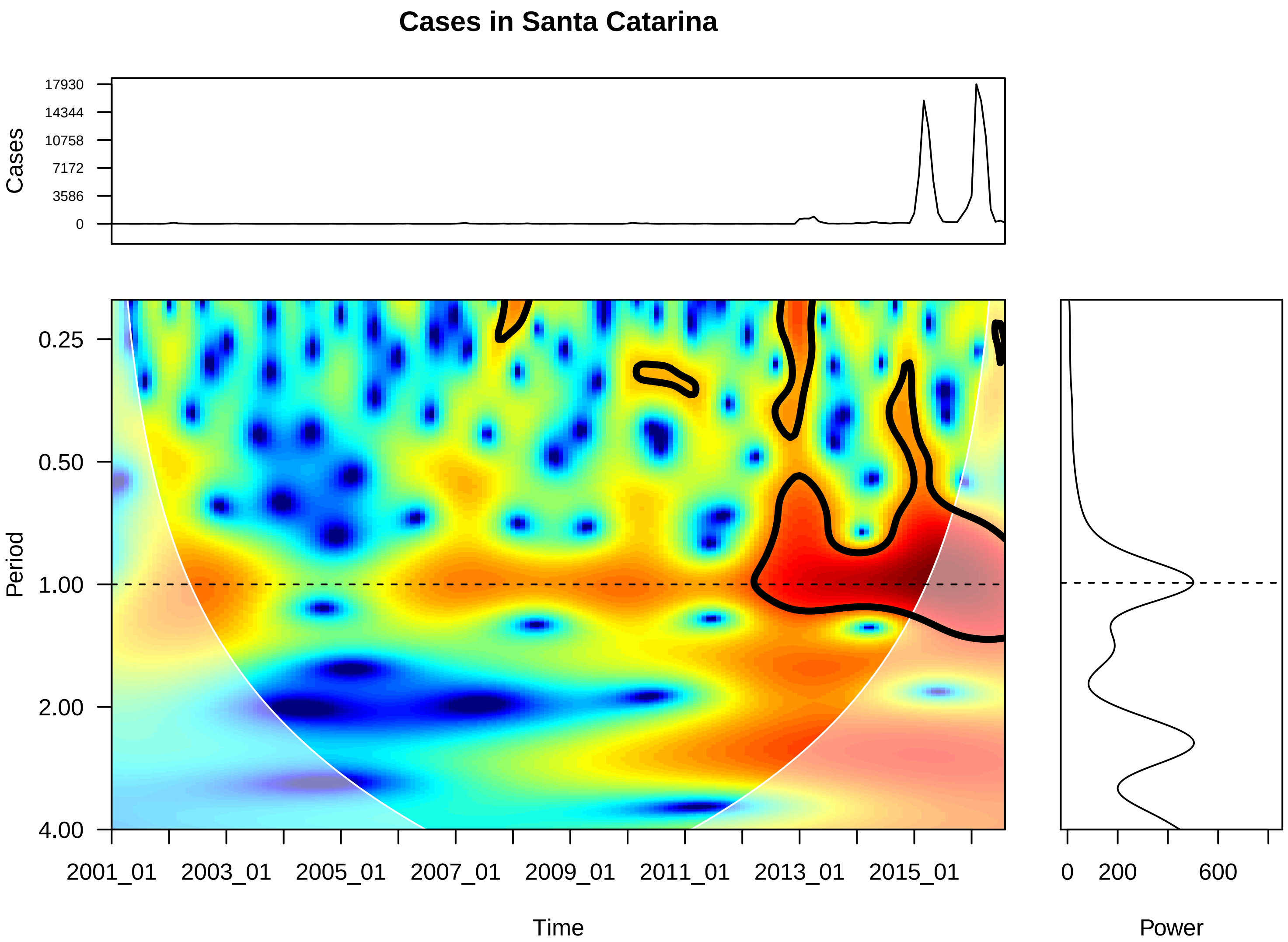

Cases in Santa Catarina
Cases
17930
14344
10758
7172
3586
0
Period
0.25
0.50
1.00
2.00
4.00
2001_01
2003_01
2005_01
2007_01
2009_01
2011_01
2013_01
2015_01
Time
0
200
600
Power

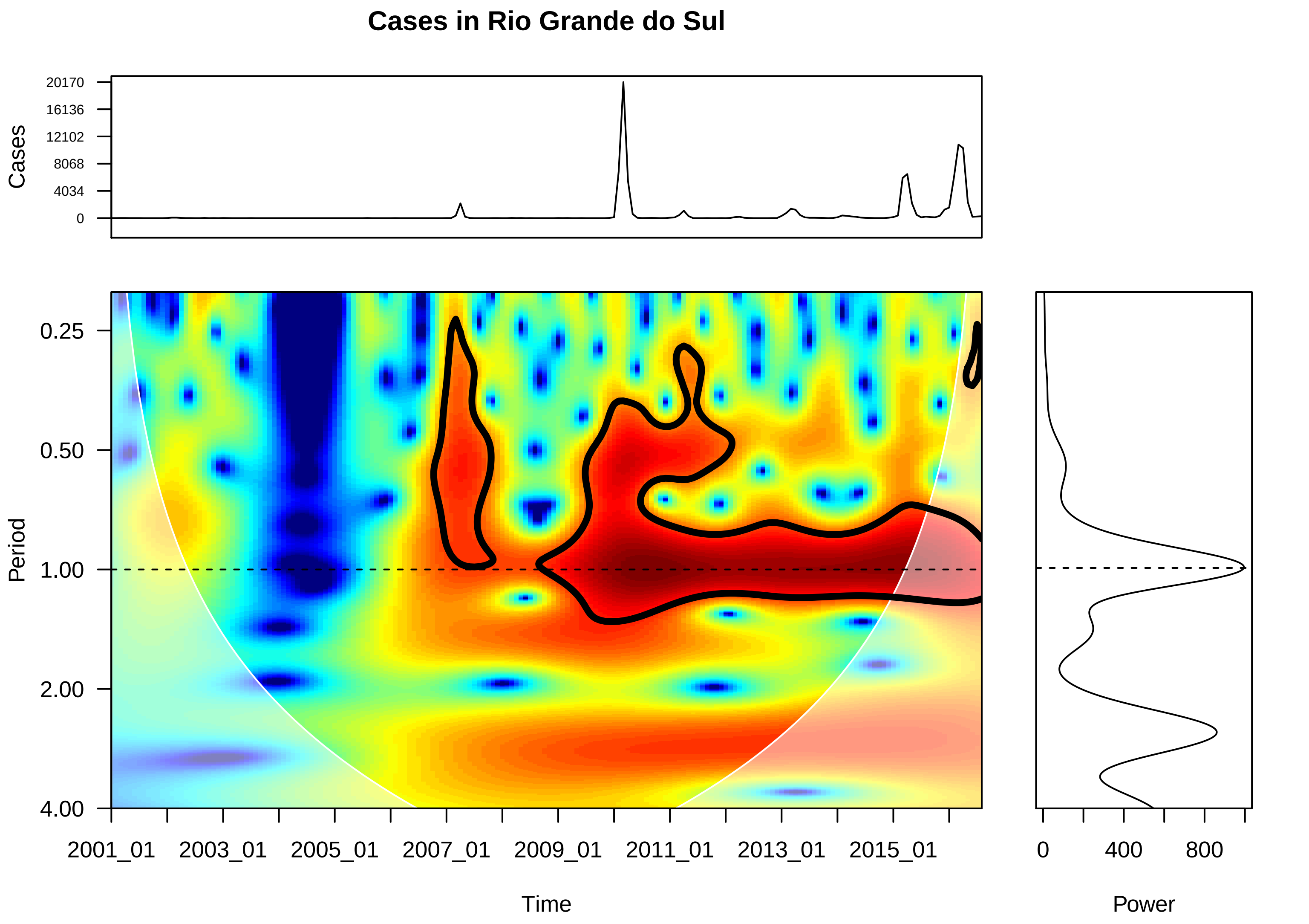
Cases in Rio Grande do Sul
Cases
20170
16136
12102
8068
4034
0
Period
0.25
0.50
1.00
2.00
4.00
2001_01
2003_01
2005_01
2007_01
2009_01
2011_01
2013_01
2015_01
Time
0
400
800
Power

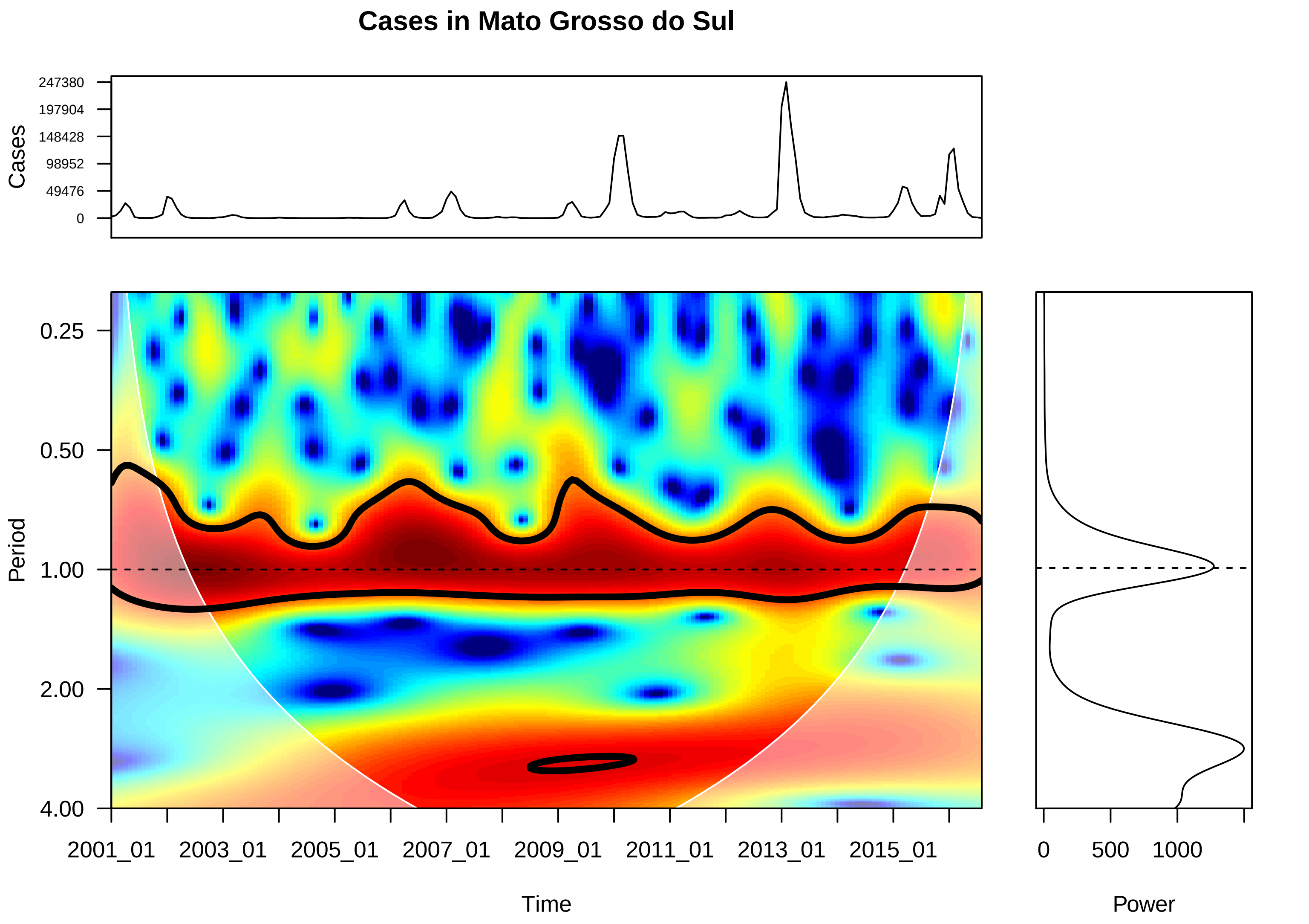

Cases in Mato Grosso do Sul
Cases
247380
197904
148428
98952
49476
0
Period
0.25
0.50
1.00
2.00
4.00
2001_01
2003_01
2005_01
2007_01
2009_01
2011_01
2013_01
2015_01
Time
0
500
1000
Power

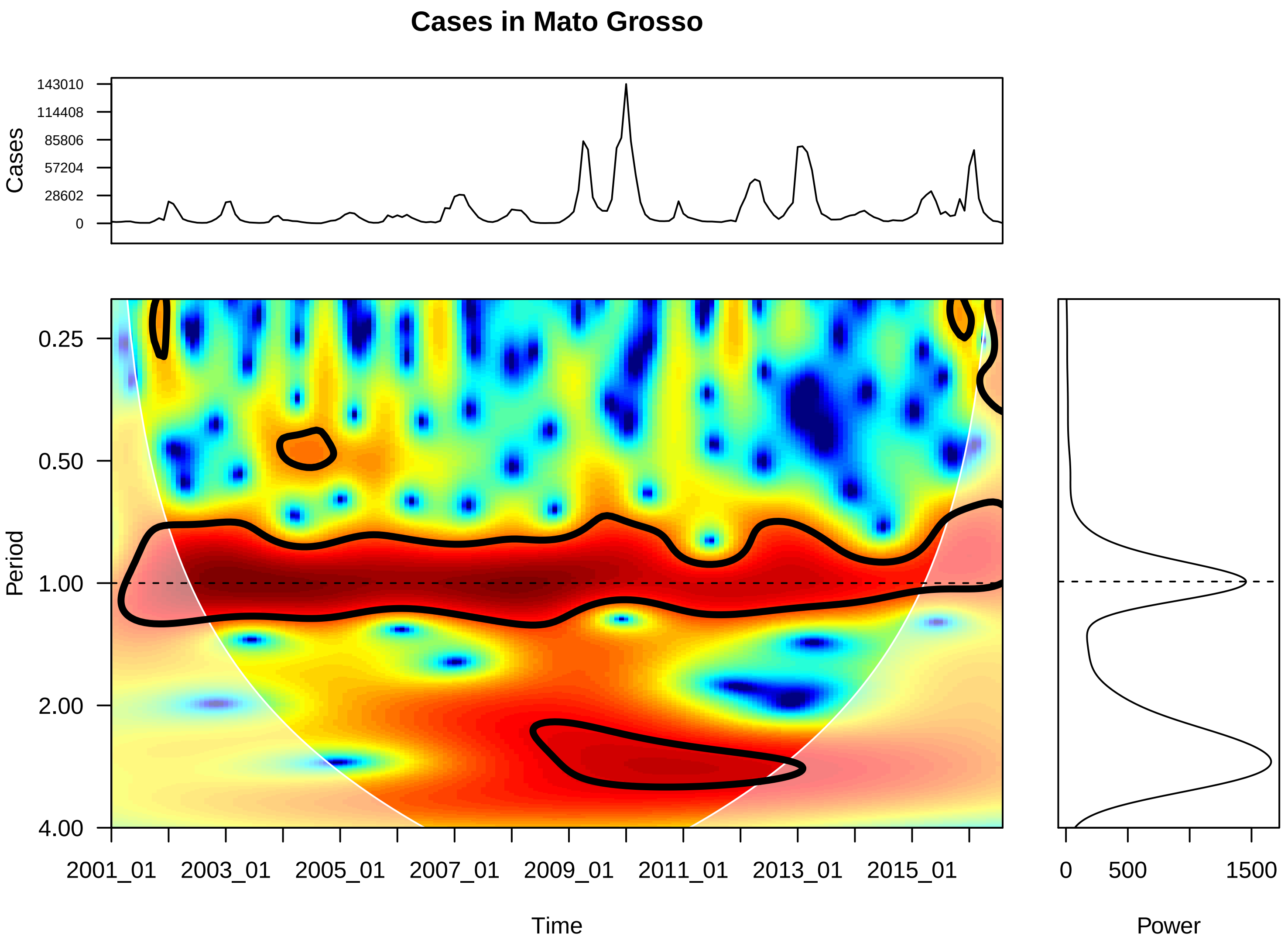

Cases in Mato Grosso
Cases
143010
114408
85806
57204
28602
0
Period
0.25
0.50
1.00
2.00
4.00
2001_01
2003_01
2005_01
2007_01
2009_01
2011_01
2013_01
2015_01
Time
0
500
1500
Power

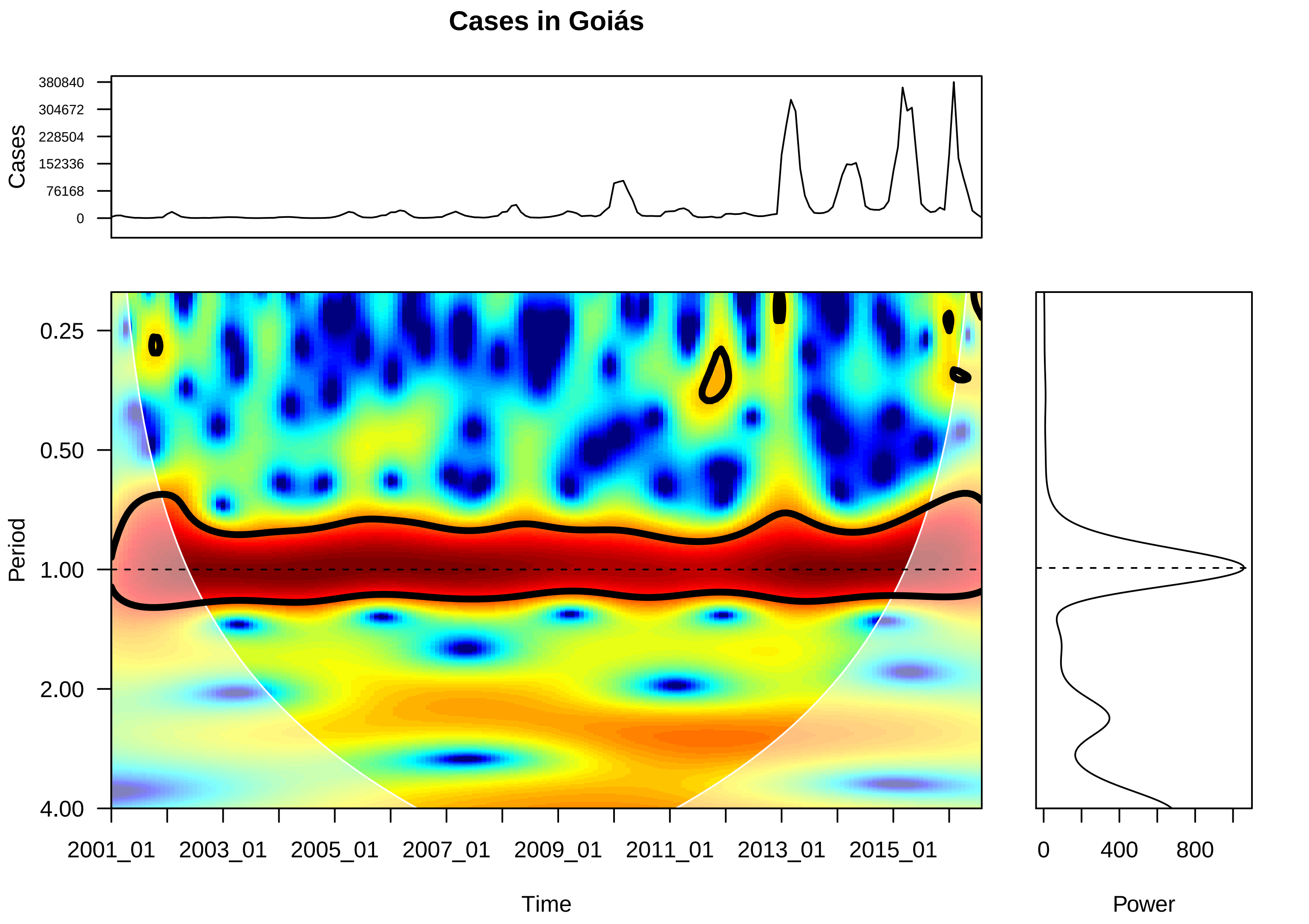

Cases in Goiás
Cases
380840
304672
228504
152336
76168
0
Period
0.25
0.50
1.00
2.00
4.00
2001_01
2003_01
2005_01
2007_01
2009_01
2011_01
2013_01
2015_01
Time
0
400
800
Power

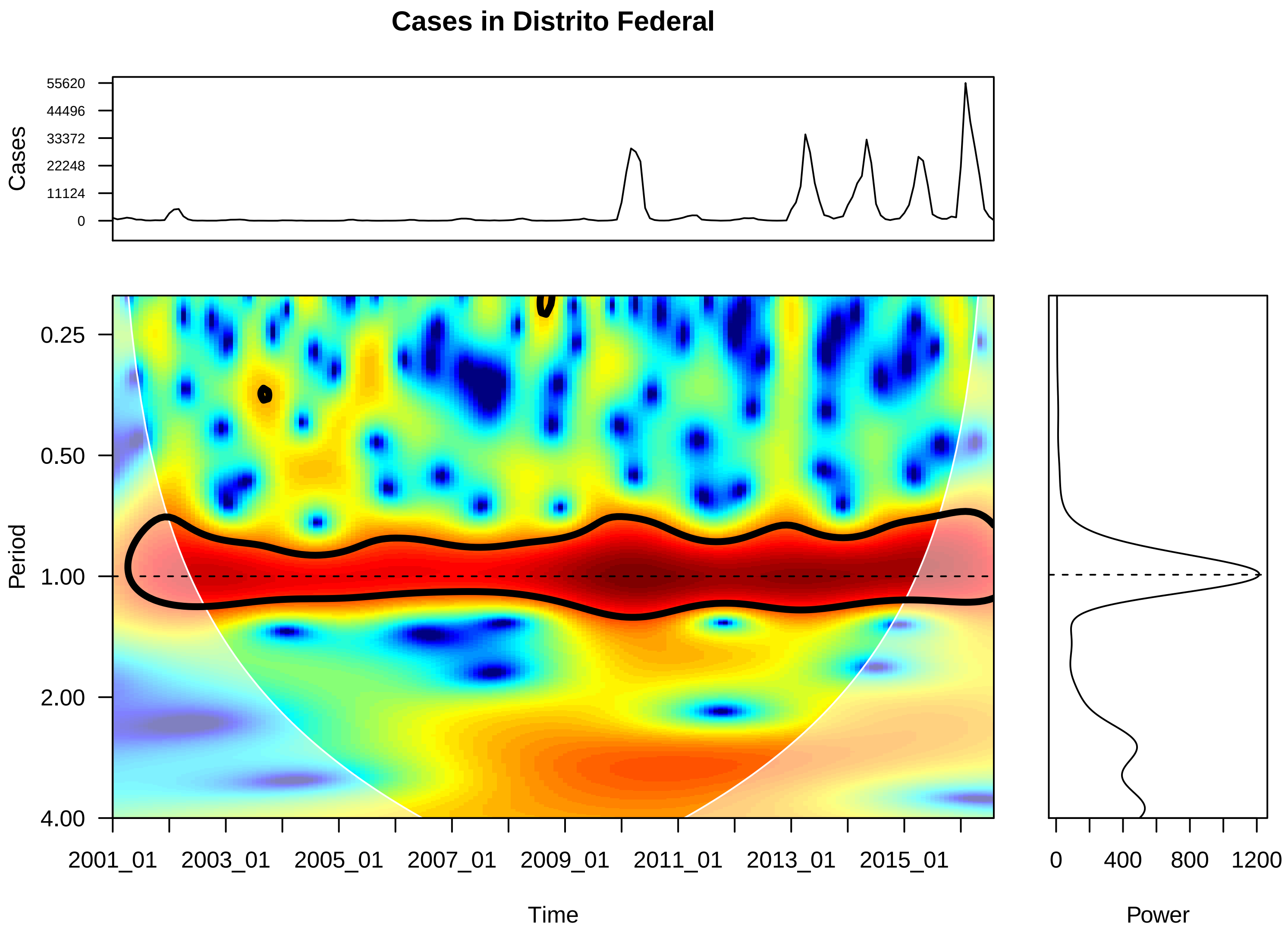
Cases in Distrito Federal
Cases
55620
44496
33372
22248
11124
0
Period
0.25
0.50
1.00
2.00
4.00
2001_01
2003_01
2005_01
2007_01
2009_01
2011_01
2013_01
2015_01
Time
0
400
800
1200
Power
