# Supplementary figures and images for: Spatio-temporal dynamics of dengue in Brazil: Seasonal travelling waves and determinants of regional synchrony
Source: PLoS Negl Trop Dis. 2019 Apr 22;13(4):e0007012. doi: 10.1371/journal.pntd.0007012 (PMC6497439; doi:10.1371/journal.pntd.0007012)

Phase lag from other regions

2  
1  
0  
-1  
-2  
-3

0

50

100

150

Time

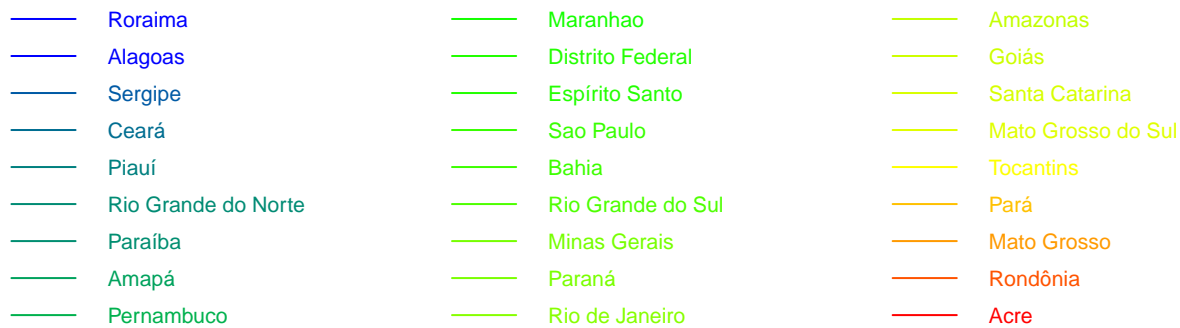

Supplement: S2 Fig — Roraima and Amapá were the least consistent while other states had similar phase lags throughout the study period. (PDF) [file pntd.0007012.s002.pdf]

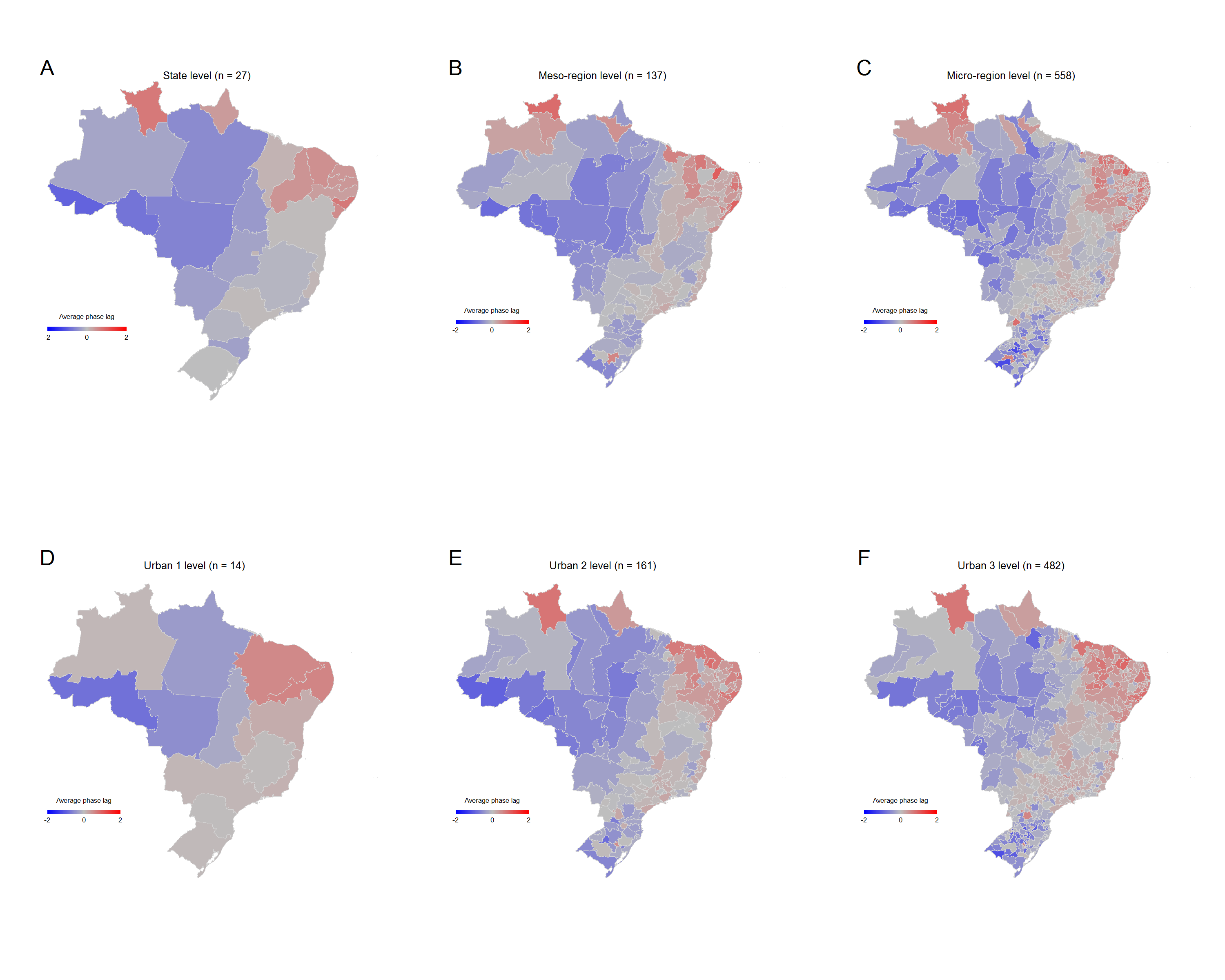

Supplement: S3 Fig — (A) state level, (B,C) meso- and micro-regions, (D,E,F) urban subdivisions. Administrative boundaries for Brazilian municipalities were obtained from IBGE (https://www.ibge.gov.br/geociencias-novoportal/cartas-e-mapas.html), along with shapefiles for Urban-Regional divisions (https://ww2.ibge.gov.br/home/geociencias/geografia/default_divisao_urbano_regional.shtm). (PNG) [file pntd.0007012.s003.png]

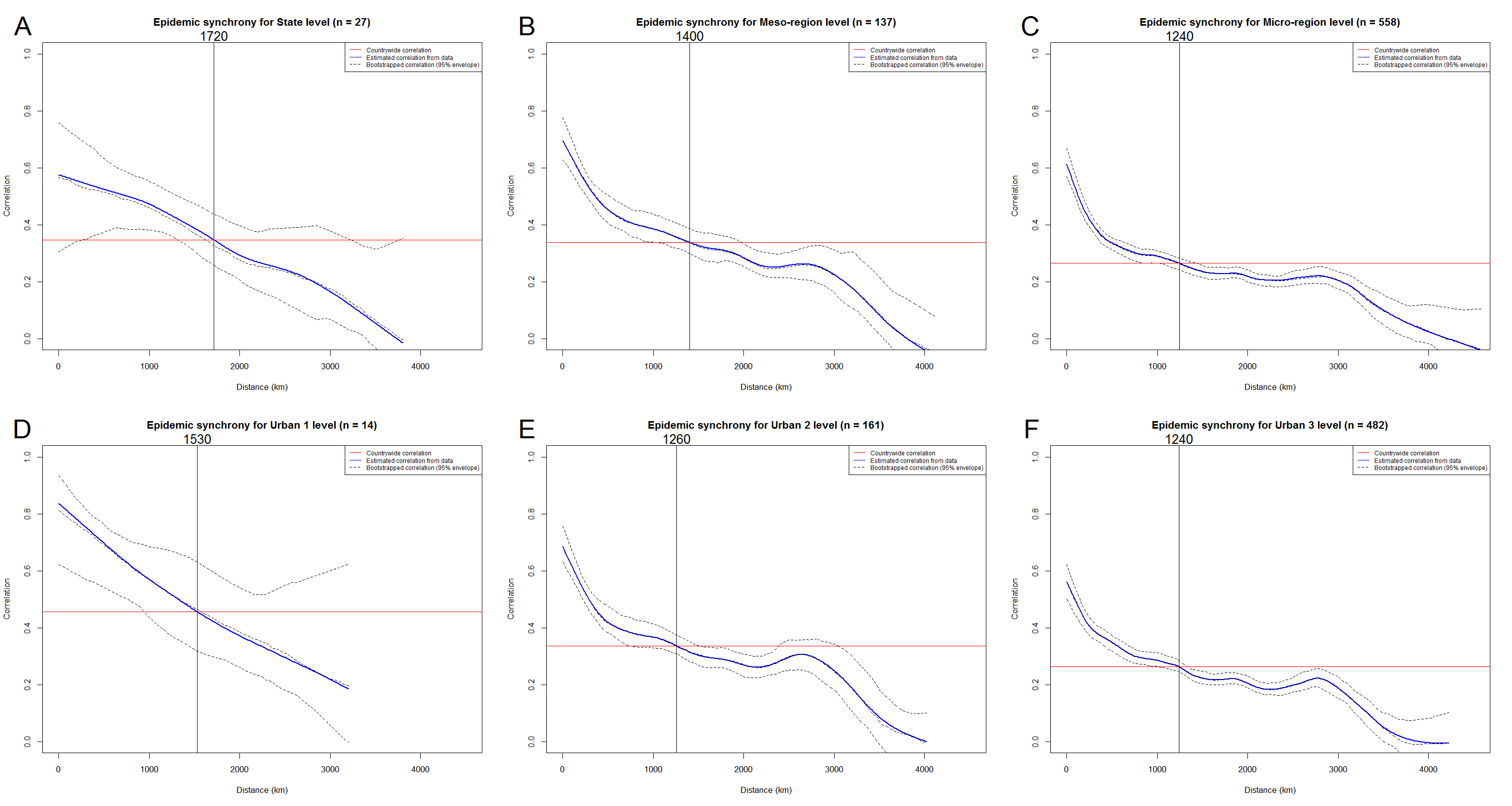

Supplement: S4 Fig — (A) state level, (B,C) meso- and micro-regions, (D,E,F) urban subdivisions. (PNG) [file pntd.0007012.s004.png]

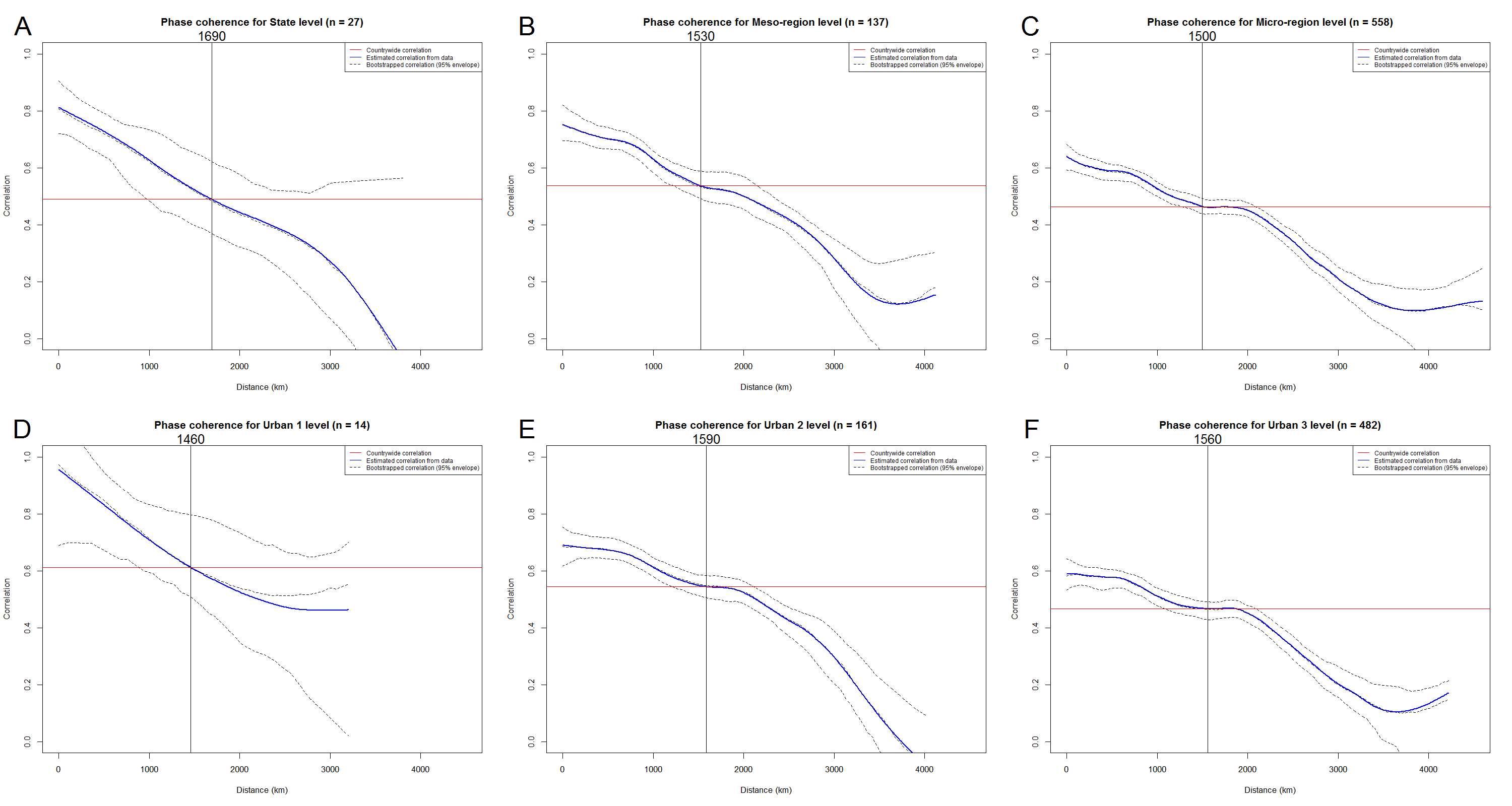

Supplement: S5 Fig — (A) state level, (B,C) meso- and micro-regions, (D,E,F) urban subdivisions. (PNG) [file pntd.0007012.s005.png]
